# Potential Therapeutic Roles of Icariin in Inflammatory Diseases: From Molecular Mechanisms to Clinical Translation Prospects

**DOI:** 10.3390/antiox15091068

**Published:** 2026-08-26

**Authors:** Sirui Du, Weifeng Li, Meixiu Jiang

**Affiliations:** 1 School of Pharmacy, Jiangxi Medical College, Nanchang University, 999 Xuefu Road, Nanchang 330031, China; 4209122065@email.ncu.edu.cn (S.D.); 4209123010@email.ncu.edu.cn (W.L.); 2 Jiangxi Province Key Laboratory of Bioengineering Drugs, The National Engineering Research Center for Bioengineering Drugs and the Technologies, Institute of Translational Medicine, Jiangxi Medical College, Nanchang University, 999 Xuefu Road, Nanchang 330031, China

**Keywords:** icariin, inflammatory diseases, anti-inflammation, molecular mechanisms, development prospects

## Abstract

Inflammatory diseases are caused by overactivation of the immune system and lead to tissue damage; their high incidence and complications seriously threaten human health. Currently, non-steroidal anti-inflammatory drugs (NSAIDs) and immunosuppressants are the mainstay of clinical practice, but their long-term use can cause gastrointestinal damage, drug resistance, and other problems. In recent years, traditional Chinese medicine has become a research hotspot for the treatment of inflammatory diseases, among which Icariin (ICA), the main active ingredient of Epimedium, has demonstrated significant anti-inflammatory potential. In this review, a comprehensive literature search of PubMed and Web of Science was conducted, and original studies on the anti-inflammatory effects and mechanisms of ICA were included. The roles and mechanisms of ICA in inflammatory diseases in different systems are summarized. Specifically, ICA suppresses NF-κB and MAPK signaling, inhibits NLRP3 inflammasome activation, and activates the Nrf2/HO-1 antioxidant axis, thereby reducing the production of pro-inflammatory cytokines such as TNF-α, IL-6, and IL-1β. Overall, the current evidence indicates broad anti-inflammatory effects of ICA across multiple organ systems, particularly in atherosclerosis and myocardial infarction; however, clinical translation is limited by poor bioavailability and the lack of standardized dosage data. Future efforts should focus on pharmacokinetic optimization and well-designed clinical trials to facilitate its application in inflammatory diseases.

## 1. Introduction

Inflammation is a complex defense response of an organism to various injurious stimuli (e.g., pathogen invasion, tissue damage, and abnormal immune response), and its essence is the process by which the body’s immune system tries to remove the harmful stimuli and initiate tissue repair. The mechanisms underlying inflammation are extremely complex and involve numerous cells and molecules, forming a dynamic regulatory network. However, in some cases, the inflammatory response can become uncontrolled, and excessive or persistent inflammation can cause serious damage to the body, triggering a range of inflammation-related diseases [1], such as multiple sclerosis, asthma, and atherosclerosis [2,3,4]. Given the severe harm caused by aberrant inflammatory responses, it is particularly critical to clarify the regulatory mechanisms underlying inflammation. In principle, the inflammatory response can be controlled at four levels, which correspond to the four components of the inflammatory pathway: inducers, receptors, mediators, and target tissues. The key control point is the production of inflammatory mediators regulated by major anti-inflammatory signals (e.g., IL-10, TGF-β, and glucocorticoids) [5].

Currently, a wide variety of drugs are used clinically for anti-inflammatory purposes, including non-steroidal anti-inflammatory drugs (NSAIDs), steroidal anti-inflammatory drugs (glucocorticoids), disease-modifying anti-rheumatic drugs (DMARDs), and biologics. NSAIDs have anti-inflammatory, antipyretic, and analgesic effects and are widely used to relieve mild to moderate pain and inflammation [6], but their side effects are serious, including gastric mucosa and small intestine injuries, cardiovascular disease, kidney injury, hepatotoxicity, and cerebral hemorrhage [7]. Short-term use of glucocorticoids is usually associated with mild side effects, including skin reactions, hypertension, hyperglycemia, hematologic abnormalities, immunosuppression, and neuropsychiatric symptoms. Long-term use of glucocorticoids may result in aseptic osteonecrosis, adrenal insufficiency, gastrointestinal and hepatic damage, hyperlipidemia, reduced growth, and even congenital malformations [8]. DMARDs are a class of medications used in the treatment of many types of arthritis, with limitations regarding anti-inflammatory specificity. The toxicity profiles of different DMARDs vary significantly, and the stage of treatment at which most adverse effects occur varies between drugs [9]. The emergence of biologics has brought new hope for the treatment of inflammatory conditions and is now being used in the treatment of diseases such as ulcerative colitis (UC) [10], especially providing a new option for patients who have failed to respond to or have had poor outcomes from treatment with traditional antirheumatic drugs. As inflammatory arthritis is a systemic disease, it is often associated with multiple extra-articular symptoms and systemic complications. Therefore, the potential effects of drugs on extra-articular symptoms or systemic complications must be comprehensively considered when selecting appropriate bDMARDs for individual patients [11]. Moreover, owing to their limited application, reasonable doses, durations, long-term clinical efficacies, and safety issues of these treatments have yet to be investigated [12]. Against this backdrop, existing reviews have indicated that natural active ingredients derived from Chinese medicinal herbs serve as high-quality research resources for developing novel anti-inflammatory drugs with low toxicity [13]. Therefore, searching for novel anti-inflammatory agents from traditional Chinese medicines is of clinical significance and social value.

**Figure 1:**
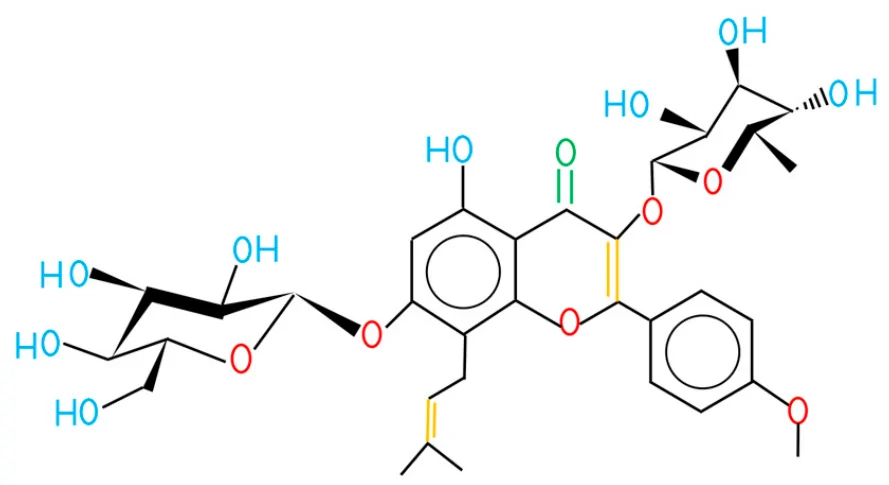
Structural features of ICA that contribute to its bioactivity include phenolic hydroxyl groups (antioxidant), prenyl moiety (membrane affinity), and flavonoid backbone (scaffold for receptor interaction). In the structure, blue highlights indicate hydroxyl groups, red indicates oxygen atoms, green indicates the carbonyl oxygen, and orange indicates carbon–carbon double bonds.

Epimedium is a genus of perennial plants belonging to the family Berberidaceae rather than a single plant species. Several Epimedium species have been established as natural sources of ICA, including *E. brevicornum* Maxim., *E. sagittatum* (Sieb. et Zucc.) Maxim., *E. pubescens* Maxim., *E. koreanum* Nakai, *E. wushanense* T.S. Ying, *E. grandiflorum* C. Morren, *E. acuminatum* Franch., and *E. pseudowushanense* B.L. Guo [14,15]. Among these species, *E. brevicornum*, *E. sagittatum*, *E. pubescens*, *E. koreanum*, and *E. wushanense* are particularly well-characterized botanical sources of ICA and related prenylated flavonol glycosides. Although several other medicinal plants, such as *Cuscuta chinensis*, *Drynaria fortunei*, *Polygonum multiflorum,* and *Ligustrum lucidum*, are frequently used for similar therapeutic indications, current phytochemical evidence does not establish them as major natural sources of ICA. Therefore, the present review focuses on ICA derived predominantly from Epimedium spp. ICA is a structurally defined flavonol glycoside (Figure 1) isolated from Epimedium and is one of the main components of Epimedium that exerts various pharmacological activities [16]. Its chemical name is 4′-O-methyl-8-γ,γ-dimethylallyl kaempferol-3-rhamnoside-7-glucoside, with molecular formula C_33_H_40_O_15_ and molecular weight 676.668.

**Figure 2:**
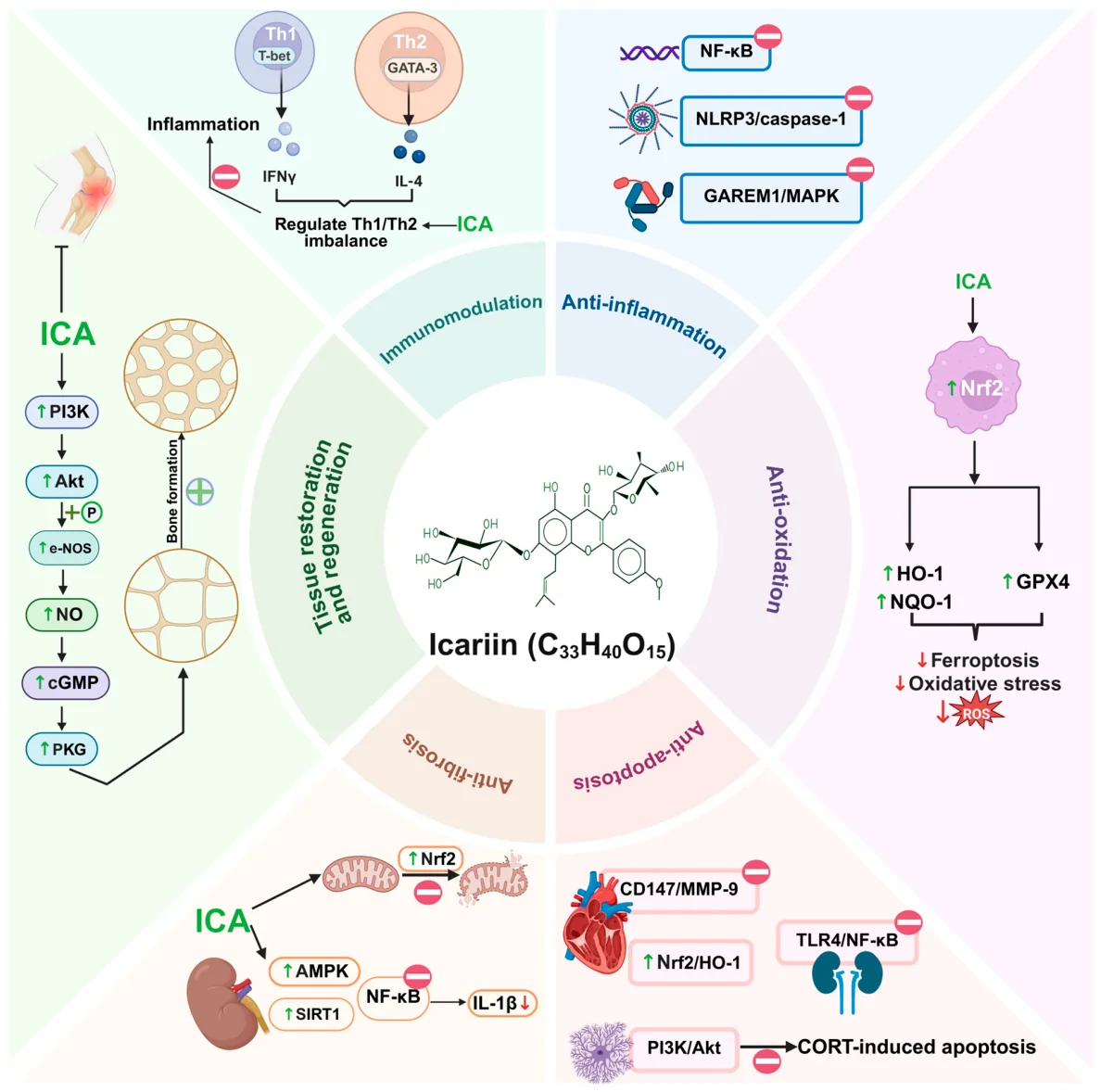
Pharmacological network of ICA. The diagram depicts ICA (C_33_H_40_O_15_) as a convergent multi-target agent that simultaneously engages six functionally interconnected regulatory modules: immunomodulation (restoring the Th1/Th2 balance via T-bet and GATA-3), tissue restoration and regeneration (activating the PI3K/AKT/eNOS/NO/cGMP/PKG cascade to promote bone formation), anti-inflammation (suppressing NF-κB, NLRP3/caspase-1, and GAREM1/MAPK signaling), anti-oxidation (activating the Nrf2/HO-1/NQO-1 axis and GPX4 to attenuate ROS, oxidative stress, and ferroptosis), anti-apoptosis (inhibiting CD147/MMP-9 and TLR4/NF-κB while activating Nrf2/HO-1 and PI3K/AKT), and anti-fibrosis (activating AMPK and SIRT1, suppressing NF-κB and IL-1β, and protecting mitochondria via Nrf2). Rather than acting on isolated pathways, ICA achieves coordinated multi-target homeostasis through cross-reinforcing signaling, in which Nrf2-dependent HO-1 induction attenuates NF-κB transcriptional activity while PI3K/AKT signaling concurrently promotes eNOS-dependent protection. Green upward arrows (↑) indicate activation or upregulation by ICA; red prohibition symbols (Ө) and T-shaped bars (⊥) indicate inhibition or blockade by ICA; the plus-in-circle symbol (⊕) indicates promotion or positive regulation; red downward arrows (↓) indicate downregulation; “+P” indicates phosphorylation; solid arrows indicate signal transduction. Abbreviations: AKT, protein kinase B; AMPK, AMP-activated protein kinase; caspase-1, cysteine-aspartic protease 1; CD147, cluster of differentiation 147 (basigin); cGMP, cyclic guanosine monophosphate; eNOS, endothelial nitric oxide synthase; GAREM1, GRB2-associated regulator of MAPK1; GATA-3, GATA binding protein 3; GPX4, glutathione peroxidase 4; HO-1, heme oxygenase-1; ICA, icariin; IFNγ, interferon-γ; IL-1β, interleukin-1β; IL-4, interleukin-4; MAPK, mitogen-activated protein kinase; MMP-9, matrix metalloproteinase-9; NF-κB, nuclear factor-κB; NLRP3, NOD-, LRR-, and pyrin domain-containing protein 3; NO, nitric oxide; NQO-1, NAD(P)H quinone oxidoreductase 1; Nrf2, nuclear factor erythroid 2-related factor 2; PI3K, phosphoinositide 3-kinase; PKG, protein kinase G; ROS, reactive oxygen species; SIRT1, silent information regulator 1; T-bet, T-box transcription factor TBX21; Th1, T helper type 1 cell; Th2, T helper type 2 cell; TLR4, toll-like receptor 4. Created in BioRender. Weifeng, L. (2026) https://BioRender.com/h8m4v7x.

The study of ICA’s anti-inflammatory effect not only helps to reveal its pharmacological mechanism and provide a theoretical basis for its clinical application but may also provide new ideas and lead compounds for the development of new anti-inflammatory drugs. Studying ICA’s anti-inflammatory mechanism is expected to provide a new direction for solving the problems of the current anti-inflammatory drugs and bring new breakthroughs in the treatment of inflammation-related diseases (Figure 2).

Despite the growing body of evidence on the anti-inflammatory effects of ICA, existing reviews have largely focused on individual organ systems or single signaling pathways; a comprehensive synthesis that integrates the molecular mechanisms of ICA across different inflammatory diseases, together with a critical assessment of the barriers to clinical translation, is still lacking. To fill this gap, the present review systematically summarizes ICA’s roles and mechanisms in inflammatory diseases of the respiratory, cardiovascular, neurologic, digestive, urinary, and motor systems; highlights the quantitative pharmacological data and translational bottlenecks (e.g., poor bioavailability and the lack of standardized dosage regimens); and proposes potential strategies to overcome these limitations, thereby providing a practical roadmap for future research and clinical application of ICA.

A comprehensive literature search was performed in PubMed and Web of Science to identify relevant studies published from January 1996 to December 2025. The following search terms were used: (icariin OR icaritin OR “icariside II”) AND (inflammation OR “inflammatory disease” OR “anti-inflammatory”). Original research articles reporting the anti-inflammatory effects, mechanisms, or clinical applications of ICA were included. Reviews, conference abstracts, editorials, and articles not published in English were excluded. The titles and abstracts of all retrieved records were screened, and the full texts of potentially relevant articles were assessed for eligibility.

## 2. The Roles and Mechanisms of ICA in Inflammatory Diseases

### 2.1. The Role of ICA in the Respiratory System

**Figure 3:**
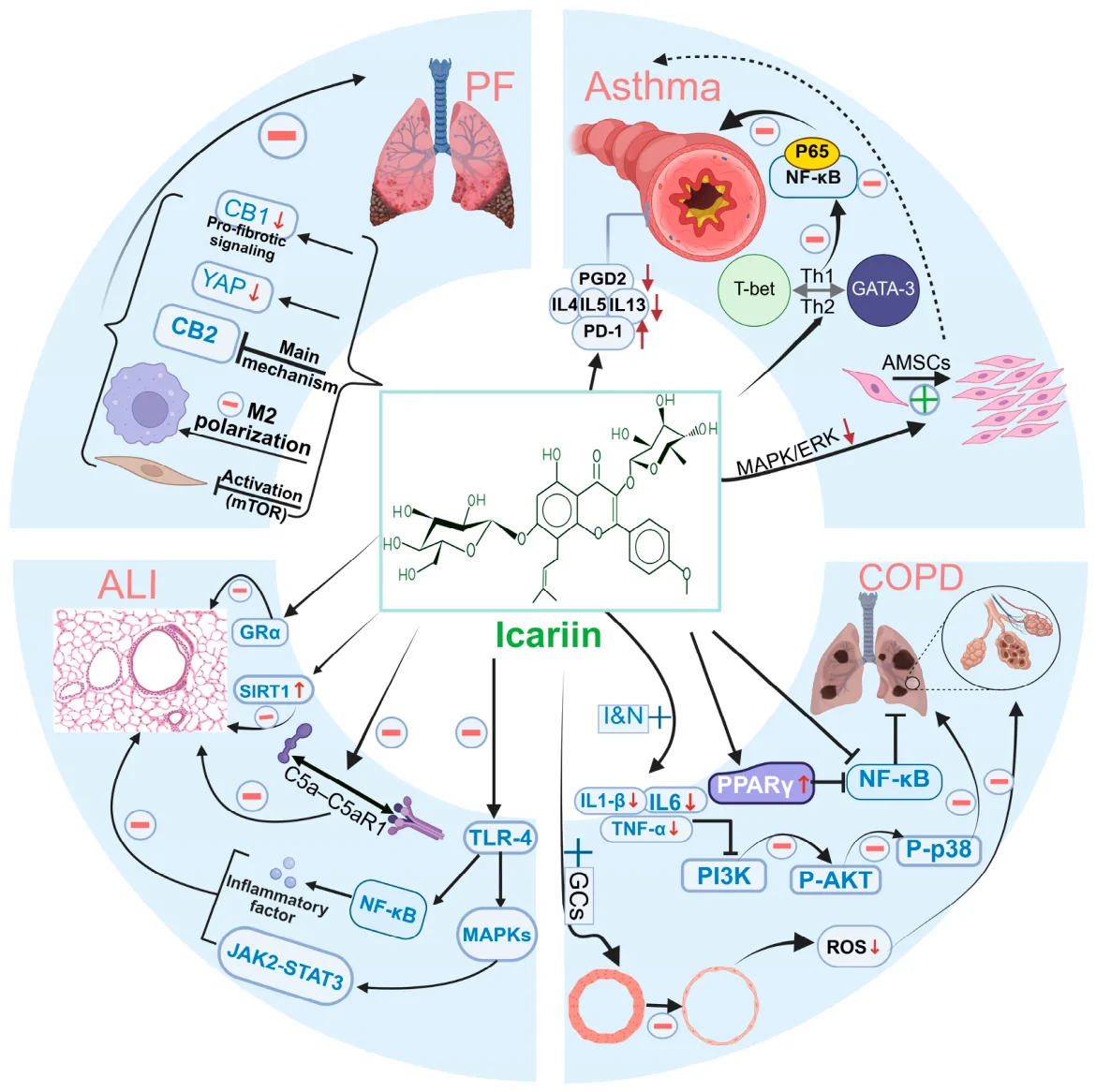
Multi-system regulatory network of ICA in respiratory inflammatory diseases. The diagram depicts the multidimensional regulatory network of ICA in four major respiratory diseases. In pulmonary fibrosis (PF), ICA downregulates YAP and CB1 signaling (the latter being pro-fibrotic), acts as a CB2 receptor antagonist (considered the main underlying mechanism), inhibits M2 macrophage polarization, and suppresses fibroblast activation via mTOR signaling. In asthma, ICA restores the Th1/Th2 balance and inhibits NF-κB and MAPK/ERK signaling, as well as airway smooth muscle proliferation, while upregulating PD-1. In chronic obstructive pulmonary disease (COPD), ICA upregulates PPARγ, which, in turn, suppresses NF-κB signaling and attenuates PI3K/AKT/p38 signaling and ROS production, with synergistic enhancement of glucocorticoid sensitivity. In acute lung injury (ALI), ICA blocks the complement C5a–C5aR1 axis and the TLR4-mediated NF-κB/MAPKs/JAK2-STAT3 signaling pathway, while upregulating SIRT1 and modulating GRα. Together, these actions reveal the pharmacological mechanism and translational potential of ICA based on multi-target intervention in respiratory diseases. Red upward arrows (↑) indicate activation or upregulation by ICA; red downward arrows (↓), T-shaped bars (⊥), and prohibition symbols (Ө) indicate inhibition or blockade by ICA; the plus-in-circle symbol (⊕) indicates promotion or positive regulation; the plus sign (+) in combined labels (e.g., “+GCS”) indicates combination or augmentation. Created in BioRender. Weifeng, L. (2026) https://BioRender.com/mukfwju.

Common respiratory diseases include pulmonary fibrosis, asthma, chronic obstructive pulmonary disease (COPD), and acute lung injury (ALI). These conditions are characterized by chronic inflammation, oxidative stress, and tissue damage, leading to significant morbidity and mortality. ICA has demonstrated significant therapeutic potential through potent anti-inflammatory and anti-oxidative effects (Figure 3).

#### 2.1.1. Pulmonary Fibrosis

Pulmonary fibrosis (PF) is a chronic, progressive tissue repair response that ultimately leads to irreversible scarring and structural remodeling of the lungs, resulting in alveolar destruction and irreversible loss of lung function, with high mortality rates and limited treatment options [17]. There are still no effective interventions to stop or cure PF, and only two drugs, pirfenidone and nidazanib, are approved by the FDA for the treatment of PF; however, the cost of treatment is high and the low solubility and specificity of fibrosis-related therapeutic agents affect their efficacy [18]. Clinical trials have reported significant gastrointestinal and cutaneous adverse effects associated with pirfenidone, and nintedanib has been associated with causing diarrhea [19]. Researchers are focused on finding therapeutically effective, affordable, and highly bioavailable drugs.

Du et al. [20] found that ICA, administered orally at 60 mg/kg/day, alleviates bleomycin (BLM)-induced PF in rats by suppressing inflammatory responses, inhibiting extracellular collagen deposition, reducing fibrotic activity, and downregulating YAP expression; therapeutic ICA was given from days 8 to 28 after BLM administration, whereas the prophylactic-plus-therapeutic regimen began 3 days before BLM exposure and continued to day 28. The authors also demonstrated that cannabinoid receptor 1 (CB1) and cannabinoid receptor 2 (CB2) were elevated in the lung tissues of fibrotic rats and that ICA decreased the expressions of both receptors; notably, CB1 overactivity contributes to the pathogenesis of PF, whereas targeting CB2, with ICA acting as a CB2 receptor antagonist, may be the main underlying mechanism of the anti-fibrotic effect of ICA [21]. In addition to the Hippo/YAP and cannabinoid receptor pathways, ICA and its metabolite icariside II have been reported to attenuate BLM-induced PF by modulating macrophage polarization, including the inhibition of M2-like polarization and macrophage infiltration [22,23]. Moreover, the effective compounds of the Jinshui Huanxian formula, including ICA, ameliorate fibroblast activation in PF by inhibiting mTOR signaling [24], and inhalable formulations of ICA have been developed to improve its pulmonary delivery for PF treatment [25]. These findings suggest that ICA may serve as an effective therapeutic candidate for PF.

#### 2.1.2. Asthma

Asthma is a disease with diverse clinical phenotypes, inflammatory responses, and airway remodeling caused by heterogeneous and complex gene–environment interactions. Allergen sensitization and multisystem comorbidities are the main features. However, we have been using a one-size-fits-all treatment strategy with the uniform use of corticosteroids and bronchodilators in all symptomatic patients [26]. As a chronic inflammatory disease, it requires regular treatment with anti-inflammatory drugs, such as inhaled corticosteroids, to prevent further attacks. Asthma is a heterogeneous disease with multiple underlying pathogenic mechanisms, and achieving precise interventions is key to providing personalized treatment options for patients with asthma. Several other potentially important new therapies that are attracting attention include immuno-anti-inflammatory and herbal medicines.

Several studies have confirmed the significant effects of ICA on ovalbumin (OVA)- and respiratory syncytial virus (RSV)-induced asthma and airway inflammation. Wei [27] found that oral ICA at 25, 50, and 100 mg/kg attenuated chronic OVA-induced airway inflammation and airway hyperresponsiveness in mice, with the anti-inflammatory effect associated with suppression of Th17 responses and enhancement of Treg function. Qiao [28] also showed that ICA, administered intraperitoneally at 2.5 mg per mouse in 200 μL normal saline once weekly for two consecutive weeks, reduced PGD2 concentrations in serum and bronchoalveolar lavage fluid (BALF), decreased CRTH2 mRNA expression in lung tissue, and attenuated leukocyte infiltration and airway inflammation in an OVA-sensitized and RSV-infected asthma mouse model. Furthermore, Hu [29] showed that oral ICA at 25, 50, and 100 mg/kg, administered 1 h before each OVA challenge from days 16 to 43, dose-dependently attenuated airway remodeling and inflammation, while ICA at 5, 10, and 100 μM for 24 h inhibited airway smooth muscle cell (ASMC) proliferation through suppression of the MAPK/ERK signaling pathway. Additionally, Zhu [30] demonstrated that oral ICA at 25, 50, and 100 mg/kg/day for 7 consecutive days during an OVA challenge attenuated asthmatic airway inflammation; reduced total leukocyte, eosinophil, and lymphocyte accumulations in BALF; suppressed M1 alveolar macrophage polarization; and enhanced the M2 phenotype, with the high-dose ICA group showing a particularly pronounced reduction in serum IgE. Moreover, Fu [31] showed that intragastric ICA at 10, 20, and 40 mg/kg/day from days 15 to 49 reduced IL-4, IL-13, and IL-5 levels and decreased neutrophil, eosinophil, and macrophage numbers in BALF in OVA/RSV-induced asthmatic mice, while alleviating oxidative stress, reducing apoptosis, and increasing Programmed Death Protein-1 (PD-1) expression; the authors subsequently selected 20 mg/kg for their mechanistic PD-1 experiments because it retained efficacy while avoiding disadvantages associated with the higher dose. In addition, ICA significantly alleviated refractory asthma-associated inflammatory and neuronal injury by suppressing endoplasmic-reticulum-stress-related proteins and NF-κB signaling; in the rat asthma model, ICA was administered at 10, 20, or 50 mg/kg, whereas primary fetal rat hippocampal neurons were pretreated with 10, 20, or 50 nM ICA for 2 h before exposure to 5 nM corticotropin-releasing hormone (CRH) for 24 h [32]. These findings suggest that ICA may lead to a therapeutic breakthrough and open up new therapeutic options for asthma.

#### 2.1.3. Chronic Obstructive Pulmonary Disease

Chronic obstructive pulmonary disease (COPD) is characterized by chronic, progressive lesions of the airways and lungs, with symptoms of bronchial obstruction that cannot be completely relieved using bronchodilators. Patients not only face impaired lung function but also often suffer from multiple comorbidities [33]. There is still a lack of specific treatment for COPD, and palliative regimens to improve airflow limitations remain the mainstay of treatment. During the acute exacerbation phase, drugs such as antibiotics, inhaled glucocorticosteroids, and bronchodilators are mainly used; however, the side effects of these drugs should not be ignored. Frequent use of inhaled glucocorticosteroids may lead to osteoporosis, immunosuppression, and increased risk of infections (especially the infections that precipitate an acute exacerbation of COPD). Moreover, commonly used clinical drugs, such as bronchodilators, anticholinergic drugs, and β2 agonists, may cause side effects such as heart rate disturbances, visual effects, urinary retention, and metabolic abnormalities [34].

COPD is characterized by an imbalance between pro- and anti-inflammatory factors, airway remodeling, and increased production of reactive oxygen species (ROS), which can lead to bronchial epithelial damage, inflammatory cell infiltration, and tissue remodeling [35]. Li [36] demonstrated that ICA, administered at 40 mg/kg in a COPD rat model and tested at 25, 50, and 75 μM in CSE-exposed BEAS-2B and H292 cells, attenuated airway inflammation by upregulating peroxisome proliferator-activated receptor γ (PPARγ) and suppressing NF-κB signaling. Regarding combination therapy, in vivo experiments showed that ICA combined with nobiletin (I&N), administered at a total dose of 2.12 mg/kg/day with an ICA-to-nobiletin ratio of 12.5:1, improved lung function and alleviated histopathological injury in COPD rats and reduced IL-6, IL-1β, and TNF-α levels while suppressing PI3K/AKT and p38 MAPK signaling [37]. In addition, ICA pretreatment at 20, 40, or 80 μM for 24 h before exposure to 5% cigarette smoke extract for 4 h reduced inflammatory responses, reactive oxygen species generation, and remodeling-associated changes in BEAS-2B cells and partially reversed glucocorticoid resistance, with the stronger effects generally observed at 40–80 μM [38]. Therefore, ICA has potential efficacy in the treatment of COPD and may change the existing treatment of bronchiectasis for curative conditions. However, its real therapeutic use in humans is yet to be further verified.

#### 2.1.4. Acute Lung Injury

Acute lung injury (ALI) is a severe clinical syndrome characterized by pulmonary edema and inflammation, which can be triggered by various pulmonary disease processes, often leading to severe hypoxemia requiring mechanical ventilation and predisposing patients to the development of acute respiratory distress syndrome (ARDS), which results in a high mortality rate [39]. Treatment for ALI can be divided into two main categories: supportive care and pharmacological interventions, which can be further divided into anti-inflammatory therapy and physiological function regulation therapy. However, there is still a lack of effective drugs that can significantly reduce mortality and improve patients’ quality of life. Coupled with the high morbidity and mortality rates of the disease, the development of novel and effective therapeutic agents is crucial. Natural products have attracted considerable attention in ALI therapy because of their multiple pharmacological activities. Based on evidence from ex vivo and in vivo studies, flavonoids, alkaloids, and terpenoids have significant anti-inflammatory and lung-protective effects [40].

Guo [41] pointed out that ICA, administered intragastrically at 30 or 60 mg/kg/day for 7 consecutive days before a single intraperitoneal LPS challenge (10 mg/kg), inhibited overactivation of the complement C5a–C5aR1 axis and TLR4-mediated NF-κB/MAPK/JAK2–STAT3 signaling, thereby attenuating inflammatory responses and mitochondrial apoptosis in LPS-induced acute lung injury; pulmonary inflammatory and molecular outcomes were evaluated 6 h after LPS administration. It has also been shown that ICA attenuates ALI in mice with adrenal insufficiency through the modulation of GRα [42]. In addition, ICA also plays a protective role against intestinal ischemia/reperfusion (I/R)-induced ALI through the upregulation of SIRT1 [43]. Regarding new dosage forms, Yu [44] discovered that an icariin–phospholipid complex (IPC), an aerosolized delivery method to achieve slow drug release, enhanced lung affinity and slowed lung clearance, thus improving the therapeutic effect of ICA on ALI. Collectively, although the treatment of ALI is based on emergency mechanical ventilation as the main life-sustaining means, the underlying treatment also requires ICA’s anti-inflammatory effect.

### 2.2. The Role of ICA in the Cardiovascular System

Cardiovascular diseases such as atherosclerosis, coronary heart disease and myocardial infarction, and diabetic cardiomyopathy, as well as myocardial hypertrophy and fibrosis, are often driven by inflammation and tissue remodeling. ICA effectively addresses the inflammatory and fibrotic processes underlying various cardiovascular disorders through these multi-target actions (Table 1).

#### 2.2.1. Atherosclerosis

Risk factors for atherosclerosis include hypercholesterolemia, hypertension, diabetes mellitus, and smoking, all of which are involved in the pathogenesis of the disease. However, it is widely recognized that atherosclerosis is essentially a chronic inflammation of blood vessels triggered by the interaction of the abovementioned risk factors with the arterial wall cells [45]. A variety of Western drugs have been widely used in the treatment of atherosclerosis, with antiplatelet agents, statins (HMG-CoA reductase inhibitors), fibrates (lipoprotein lipase activators), and niacin being the most commonly used medications, which are usually based on a single-target design; used according to diagnostic results; and have a variety of side effects, e.g., antiplatelet agents are prone to cause bleeding and statins, are prone to cause muscle soreness or weakness.

There are various ways for ICA to improve atherosclerosis: Regarding lipid-lowering, ICA administered orally at 30 or 60 mg/kg/day improved high-cholesterol diet (HCD)-induced dyslipidemia in rats by reducing their serum IL-6 and TNF-α levels, tissue mRNA expression, and p-p38 MAPK expression [46]. Other studies have demonstrated that ICA can also reduce serum total cholesterol and low-density lipoprotein cholesterol levels, with lipid-lowering effects, thus preventing and controlling thrombus formation during atherosclerosis [47]. Regarding arterial plaque reduction, ICA administered at 30 or 60 mg/kg/day significantly reduced arterial plaque area and macrophage infiltration by downregulating CX3CR1 expression [48]. In addition, ICA effectively improves endothelial cell dysfunction, reduces endothelial atherosclerotic plaque area and collagen fibers in aortic sinus tissue, and alleviates atherosclerotic lesions [49]. Moreover, ICA at 4 or 20 μM can inhibit foam cell formation by downregulating the expression of CD36 and upregulating the expression of SR-BI, which plays a role in inhibiting atherosclerosis [50]. In addition, in the oxidized low-density lipoprotein (Ox-LDL)-induced AS cell model, ICA may also inhibit the proliferation and migration of arterial vascular smooth muscle cells (HAVSMCs) through the upregulation of miR-205-5p, thus reducing atherosclerosis [51]. Moreover, Hu [52] demonstrated that ICA at 10, 20, or 40 μmol/L attenuated oxidative damage in human umbilical vein endothelial cells (HUVECs); inhibited monocyte adhesion to HUVECs; and decreased ICAM-1, VCAM-1, and E-selectin expression in a concentration-dependent manner. Zhang’s [53] study demonstrated that oral ICA at 20, 40, or 80 mg/kg/day for 12 weeks significantly inhibits atherosclerosis formation by lowering the levels of protein kinase CβI and its phosphorylated form in the aorta, and Fang’s [54] study further demonstrated that ICA exerts its anti-atherosclerotic effects through a variety of mechanisms: it can not only attenuate DNA damage and improve endothelial dysfunction but also inhibit the proliferation and migration of smooth muscle cells, as well as block macrophage-derived foam cell migration. The above studies show that ICA is effective at preventing atherosclerosis through the synergistic effect of multiple pathways.

#### 2.2.2. Coronary Atherosclerotic Heart Disease and Myocardial Infarction

Coronary atherosclerotic heart disease (CAD) is one of the most common causes of myocardial ischemia, the core of which is atherosclerosis of the coronary arteries supplying blood to the heart, resulting in narrowing or occlusion of blood vessels, which triggers myocardial ischemia; furthermore, myocardial infarction is one of the most serious consequences of CAD. Commonly used drugs for this type of disease include antiplatelet drugs, ester-lowering drugs, and angina drugs for the relief of morbid symptoms. Their side effects include gastrointestinal bleeding, mostly with long-term use of drugs, which cannot be stopped or adjusted without authorization. Increasingly more studies have found the advantages of Chinese medicine in the treatment of these diseases: not only can it prevent the occurrence of these diseases but also alleviate the symptoms of the disease and reduce the side effects of Western medicine in the treatment of this disease.

ICA plays a therapeutic role in coronary artery disease and myocardial infarction via multiple targets. Xin [55] found that ICA may be used to treat coronary artery disease and myocardial infarction through the action of target proteins such as IL-6, ESR1, EGFR, MAPK8, VEGFA, and CASP8. CASP8 and other target proteins are involved in the regulation of the PI3K–AKT and TNF signaling pathways, thus effectively treating CAD. Regarding myocardial infarction, Sai [56] demonstrated that oral ICA at 60 mg/kg once daily for 28 days reduced myocardial apoptosis and inflammatory responses and activated the Nrf2/HO-1 signaling pathway. Shi [57] identified another pathway that also attenuated cardiomyocyte apoptosis after myocardial infarction by reducing the apoptosis rate and inhibiting the CD147/MMP-9 pathway. Sharma [58] found that oral ICA at 5 or 10 mg/kg for 56 days exerted cardioprotective effects in isoproterenol-challenged Wistar rats by increasing cGMP and Nrf2 expressions and inhibiting the NF-κB/caspase-3 signaling pathway. In an experimental rat myocardial infarction model constructed by Jia [59], ICA at 30, 60, or 120 mg/kg/day significantly improved cardiac function and attenuated cardiac remodeling through the complex TGF-β1/Smad signaling pathway. In response to I/R injury, ICA at 2, 4, or 8 μM protected cardiomyocytes against oxidative stress and apoptosis by activating the SIRT1/FOXO1 signaling pathway, with 4 μM commonly used for subsequent mechanistic evaluation [60]. Studies [61,62,63] have also shown that postconditioning of ICA protects cardiomyocytes from I/R-induced oxidative stress injury through activation of the PI3K/AKT and eNOS pathways to protect against myocardial I/R injury. Ren [64] suggested that ICA exerts cardioprotective effects by increasing HSP20 levels in H9C2 cells, thereby improving cell proliferation and reducing I/R-induced apoptosis. To improve efficacy against myocardial infarction, Kamal [65] investigated new dosage forms—two protein-free bionic lipid nanoparticles mimicking the LDE and HDA of low-density lipoproteins (LDLs) and high-density lipoproteins (HDLs), respectively—and loaded them with the botanical drug epimedium glycoside. LDE and HDA showed superior cellular uptake in H9C2 cardiomyocytes, and HDA targeted infarcted myocardium better than LDE. In summary, ICA plays a therapeutic role in CAD and infarction through its anti-inflammatory, anti-apoptotic, and pro-repairing effects, and that new targeted agents can be used to improve the drug’s efficacy.

#### 2.2.3. Diabetic Cardiomyopathy

Diabetic cardiomyopathy (DCM) is a cardiac manifestation specific to patients with diabetes mellitus; it is characterized by left ventricular hypertrophy and diastolic dysfunction in the early stage, and progressing to an advanced stage may result in overt heart failure with reduced systolic function. DCM adversely affects the prognosis of patients, and there is currently no targeted therapy [66]. It has also been suggested that diabetes can trigger cardiomyopathy and increase the risk of heart failure independently of hypertension and coronary artery disease [67]. The main pathological manifestations include myocardial hypertrophy, myocardial fibrosis, and impaired ventricular function, which may trigger extensive myocardial necrosis. Eventually, the condition may progress to heart failure, arrhythmias, and cardiogenic shock, and in severe cases, even sudden cardiac death. The main treatment strategies now include effective control of blood glucose, regulation of other risk factors such as blood pressure and lipids, and cardiomyocyte protection. New drugs that protect the heart, slow myocardial remodeling, and reduce adverse effects need to be developed [68]. For this reason, specific medications should provide glucose-lowering, cardioprotective, and heart failure treatments while avoiding side effects such as those from glucose-lowering medications that can increase the risk of heart failure and from cardiac medications such as β-blockers that may mask hypoglycemic symptoms and other increased risks for patients.

Ni [69] found that ICA at 30 mg/kg for 16 weeks ameliorated DCM by preventing mitochondrial dysfunction through the Apelin/Sirt3 signaling pathway. Cai [70] also found that ICA at 30 or 60 mg/kg/day alleviated diabetic cardiomyopathy by improving cardiac function and suppressing cardiomyocyte pyroptosis through the AMPK–NLRP3 pathway, with stronger effects at 60 mg/kg/day. There are also special studies on type 2 diabetes mellitus (T2DM) cardiomyopathy, which is an important part of DCM, that demonstrated severe insulin resistance, left ventricular dysfunction, abnormal lipid deposition, cardiac inflammation, and fibrosis. Qiao [71] found that ICA at 60 or 120 mg/kg for 12 weeks may play a protective role against DCM by reducing the extracellular matrix proteins in cardiac tissues and by alleviating the above pathologies. Zhang [72] also found that ICA at 30 or 60 mg/kg for 8 weeks improved irregular collagen metabolism and myocardial fibrosis by inhibiting Jun and p65, as well as protected cardiac function in a type 2 diabetic rat model (T2DM). These results clearly show that ICA may provide a new direction for DCM treatment.

#### 2.2.4. Myocardial Hypertrophy and Fibrosis

Myocardial hypertrophy is classified into two types: physiological and pathological hypertrophies. Hypertrophy is an initial adaptive response of the body to physiological and pathological stimuli; however, pathological hypertrophy usually progresses to heart failure [73]. The main therapeutic agents for myocardial hypertrophy are the treatment of the disease that caused hypertrophy and the treatment of the cardiac disease caused by hypertrophy. Myocardial fibrosis is a pathological cardiac remodeling process that involves excessive collagen deposition and fibrous connective tissue proliferation in the myocardium, resulting in myocardial stiffness, decreased elasticity, and functional impairment. Long-term myocardial hypertrophy leads to ischemia and hypoxia, which activates fibroblasts to secrete collagen, gradually replacing hypertrophied cardiomyocytes and forming fibrosis. Myocardial fibrosis is treated with drugs to control the primary disease, as well as antifibrotic drugs. Myocardial hypertrophy is an early reversible compensation, whereas fibrosis is mostly irreversible. Current therapeutic medications, particularly β-blockers, may induce bradycardia and mask hypoglycemic symptoms, while Angiotensin-Converting Enzyme Inhibitors (ACEIs)/Angiotensin II Receptor Blockers (ARBs) can lead to hyperkalemia and renal impairment.

Hu found that ICA at 20 μM attenuated ISO-induced cardiomyocyte hypertrophy and prevented cell injury by enhancing autophagic flux through the AMPK/mTOR signaling pathway; higher concentrations of 60–80 μM reduced cell viability [74]. Zhou [75] demonstrated that ICA significantly repressed Ang II-induced cardiac hypertrophy and apoptosis with protective effects by inhibiting ROS-dependent JNK and p38 signaling pathways. For myocardial fibrosis, Sharma [58] found that ICA, like sildenafil, demonstrated a clear anti-fibrotic effect in the treatment of ISO-induced cardiac disease by significantly reducing the amount of collagen deposition through inhibition of the TGF-β1 signaling pathway. In addition, icariside II, a major metabolite of ICA, administered at 4, 8, or 16 mg/kg/day via gavage for 12 weeks attenuated myocardial fibrosis in spontaneously hypertensive rats by inhibiting NF-κB p65 expression and the TGF-β1/Smad2 signaling pathway [76]. Overall, ICA can simultaneously block myocardial hypertrophy and myocardial fibrosis and protect the heart through the regulation mechanisms of multiple pathways.

**Table 1:** Multi-target therapeutic mechanisms of ICA in cardiovascular diseases: pathological features, molecular pathways, and functional outcomes.

Disease	Pathological Features	Mechanisms of ICA (↑/↓)	Functional Outcomes	References
Atherosclerosis	Vascular inflammation, lipid deposition, and endothelial dysfunction	↓ IL-6, TNF-α, p-p38 MAPK, TC, LDL-C, CX3CR1, CD36, ICAM-1, and VCAM-1; ↑ SR-BI and miR-205-5p	Anti-inflammatory, lipid-lowering, plaque reduction, and endothelial protection	[43,44,45,46,47,48,49,50,51]
CAD & MI	Ischemia, apoptosis, oxidative stress, and remodeling	↑ Nrf2/HO-1, PI3K/AKT/eNOS, and SIRT1; ↓ NF-κB, Caspase-3, and TGF-β1	Anti-apoptotic, cardioprotective, and improves cardiac function	[52,53,54,55,56,57,58,59,60,61,62]
DCM	Mitochondrial dysfunction, pyroptosis, and fibrosis	↑ Apelin/Sirt3 and AMPK; ↓ NLRP3, Jun, and p65	Improves metabolism, reduces fibrosis, and anti-pyroptosis	[66,67,68,69]
Hypertrophy/Fibrosis	Collagen deposition, hypertrophy, and stiffness	↑ AMPK/mTOR; ↓ ROS, TGF-β1, Smad2, NF-κB, and p65	Anti-hypertrophic, anti-fibrotic, and cardiac protection	[71,72,73]

↓ indicates downregulation or inhibition; ↑ indicates upregulation or activation.

### 2.3. The Role of ICA in the Neurologic System

Neurological disorders, encompassing conditions such as hippocampal neuroinflammation, spinal cord injury, multiple sclerosis, neuropathic pain, traumatic brain injury, and ischemic stroke, are characterized by intricate pathological mechanisms, notably inflammation and tissue damage. ICA demonstrates significant multi-target therapeutic potential against these disorders by effectively modulating pivotal pathogenic pathways involved in their progression (Table 2).

#### 2.3.1. Hippocampal Neuroinflammation

The hippocampus is susceptible to damage from epilepsy, hypoxia, ischemia, or encephalitis, leading to memory loss and other disorders such as epilepsy, psychosis, depression, and intellectual disabilities [77]. Treatment strategies for hippocampal disorders are disease-specific, with drug selection tailored accordingly.

Liu et al. [78,79] reported that ICA improves hippocampal neuroinflammation by inhibiting HMGB1-RAGE signaling, providing neuroprotection via TLR4-XBP1s-related NF-κB signaling, and ameliorating depressive behaviors in mice. Excessive CORT induces neuronal damage, particularly in the hippocampus, reducing hypothalamic neuronal survival; ICA prevents CORT-induced apoptosis via the PI3K/AKT pathway [80] and protects hippocampal neurons by blocking p38MAPK phosphorylation [81]. Wang [82] demonstrated that ICA, administered via gavage at 60 mg/kg once daily for 7 consecutive days after surgery, reduced hippocampal inflammation, ameliorated neuronal injury, inhibited microglial activation, attenuated surgery-induced memory impairment, and improved cognitive function in aged rats. It also improved spatial learning in rats with LPS-induced cerebral dysfunction by inhibiting hippocampal TNF-α, IL-1β, and COX-2 expression [83]. Furthermore, Wang [84] found that ICA inhibited neuroinflammation by promoting microglial M2 polarization, alleviated seizure severity and mortality during the acute phase of pilocarpine-induced epilepsy, and improved chronic epileptic outcomes. Jiang [85] showed that ICA, administered intragastrically at 80 mg/kg once daily for 10 consecutive days, alleviated autistic-like behaviors and reduced hippocampal CA1 neuroinflammation by decreasing microglial activation and pro-inflammatory cytokine expression in adult BTBR mice. Additionally, ICA administered at 20 or 40 mg/kg exerted antidepressant effects in rats exposed to chronic mild stress by improving antioxidant and anti-inflammatory statuses in the brain and suppressing activation of the hippocampal NF-κB/NLRP3 inflammatory axis [86], indicating its potential for treating hippocampal neuritis.

#### 2.3.2. Spinal Cord Injury

Spinal cord injury (SCI) frequently results in severe, permanent neurological deficits due to primary and secondary injuries [87]. As primary injuries lack effective treatments, efforts concentrate on mitigating secondary injuries [88]. Current SCI therapies encompass high-dose methylprednisolone sodium succinate, surgical spinal stabilization and decompression, intensive medical management, and rehabilitation. Despite their effectiveness, innovative approaches are urgently required to repair damaged spinal cords and enhance patients’ quality of life [89].

Although SCI’s precise pathogenesis remains uncertain, mechanisms such as inflammation, oxidative stress, apoptosis, and autophagy are implicated [90]. Early high-dose ICA administration significantly enhances motor function recovery post-SCI by suppressing pro-inflammatory factors (IL-1β, TNF-α, iNOS), oxidative stress, and mitochondrial apoptotic pathways. It also diminishes mitochondrial apoptosis-related protein expression and neuronal apoptosis, protecting spinal cord tissues [91]. Feng [92] demonstrated that ICA, administered via gavage at 50 μmol/kg once daily for 7 consecutive days after an SCI, inhibited reactive astrocyte activation by suppressing YAP activity and regulating PPM1B ubiquitination and nuclear translocation, thereby reducing TGF-β/Smad signaling and facilitating functional recovery in SCI rats. Zhou [93] revealed that ICA reduces astrocyte activation, downregulates the TGF-β signaling pathway, and promotes axonal nerve lengthening and post-injury angiogenesis, improving behavioral and histological recovery in SCI rats. Additionally, ICA, administered orally at 35 μmol/kg once daily for 7 consecutive days, decreased pro-inflammatory and pro-apoptotic mediators; alleviated oxidative stress; and increased NQO-1, HO-1, and Nrf2 expressions in the injured spinal cord, thereby accelerating locomotor function recovery in SCI rats [94]. In summary, ICA provides the benefits of traditional Chinese medicine for SCI restorative treatment, featuring multi-target effects, minimal side effects, and suitability for long-term use.

#### 2.3.3. Multiple Sclerosis

Multiple sclerosis (MS), an acquired and disabling neurological disease predominantly affecting young adults, presents heterogeneous symptoms due to involvement of motor, sensory, visual, and autonomic systems, with relapses arising from focal demyelinating lesions [95]. While modern Western medicine has advanced MS treatment, immunosuppressive therapy may induce severe side effects, such as progressive multifocal leukoencephalopathy and infections [96].

Experimental autoimmune encephalomyelitis (EAE), a widely used model that mimics human MS, features complex immunopathological and neuropathological interactions [97]. ICA administered via gavage at 12.5 or 25 mg/kg/day for 42 consecutive days significantly ameliorated relapse-remitting EAE in SJL/J mice by reducing spinal cord inflammation and demyelination and suppressing inflammation-associated NF-κB, AKT, ERK1/2, p38, c-Jun, and MEK phosphorylation [98]. Moreover, ICA administered via gavage at 50, 150, or 300 mg/kg/day for 5 consecutive days after the onset of EAE symptoms exerted estrogen-like activity through ERβ, modulated hypothalamic–pituitary–adrenal axis function, and upregulated glucocorticoid receptor expression in cerebral white matter, with the higher ICA doses producing more pronounced neurological improvement [99]. Gao [100] demonstrated that ICA, administered via gavage at 7.5 or 15 mg/kg/day for 28 consecutive days after the appearance of EAE-associated behavioral signs, reduced demyelination and neuronal loss and inhibited microglial NLRP3 inflammasome-mediated neuroinflammation, thereby attenuating EAE-induced neurological pathology. Thus, if proven effective in humans, ICA could be a valuable complement to immunosuppressive treatments with significant side effects.

#### 2.3.4. Neuropathic Pain

Neuropathic pain (NP) is a disabling sensory disorder with an obscure etiology and limited precise therapeutic options [101]. Current treatments primarily provide symptomatic relief, encompassing non-pharmacological, pharmacological (e.g., antidepressants, anticonvulsants [102]), and interventional strategies. Chinese medicine alleviates NP through multiple pathways, including anti-inflammatory, antioxidant, anti-apoptotic, autophagy-regulating, intestinal-flora-modulating, and ion-channel-influencing mechanisms [103]. Gui [104] found that intragastric ICA at 25, 50, or 100 mg/kg/day from days 8 to 15 after paclitaxel treatment attenuated paclitaxel-induced neuropathic pain in a SIRT1-dependent manner; notably, repeated administration of 100 mg/kg significantly alleviated long-term mechanical allodynia, whereas 50 and 100 mg/kg suppressed selected spinal inflammatory responses. Pokkula [105] demonstrated that ICA reduces nerve-injury-induced inflammation and apoptosis by downregulating TNF-α, IL-6, and Bax while upregulating Bcl-2. Additionally, it inhibits NR2B, TRPV1, and Cav2.2 receptors, enhancing anti-apoptosis, suppressing inflammation, and exerting neuroprotective effects against NP. Overall, ICA is an effective NP therapeutic agent with triple neuroprotective mechanisms.

#### 2.3.5. Traumatic Brain Injury

Traumatic brain injury (TBI) is acquired brain damage caused by external mechanical forces, potentially leading to temporary or permanent dysfunction [106]. Current drug therapies for TBI primarily focus on symptomatic supportive treatment, such as lowering intracranial pressure, relieving pain, alleviating irritability, and controlling infections, with no drug definitively proven to have specific efficacy for TBI.

Du [107] discovered that ICA significantly improved neurobehavioral function and mitigated pathological injury in TBI rats. It reduced the protein expression of the inflammatory factors COX-2, IL-1β, and TNF-α, along with their regulatory proteins p-NF-κB-p65, p-ERK1/2, p-JNK, and p-p38, in the TBI-injured cortex. Additionally, another study indicated that ICA improved cognitive deficits in mice post-TBI by enhancing acetylation levels in the hippocampus [108]. These findings suggest that ICA offers a promising new direction for research on non-specific drugs for TBI.

#### 2.3.6. Ischemic Stroke

Ischemic stroke (IS) causes temporary or permanent tissue perfusion insufficiency, leading to neurovascular system damage, disrupting brain homeostasis, and triggering long-term motor and cognitive dysfunction [109]. Currently, there is a scarcity of effective drugs for comprehensive IS treatment, creating an urgent need to develop drugs targeting post-stroke pathological processes.

Zheng [110] found that ICA at 10, 20, or 40 mg/kg significantly improved neurological deficits, cerebral edema, and pathological injury after 2 h of transient MCAO followed by 24 h of reperfusion, increased neuronal survival, inhibited microglial activation and IL-1β expression, and attenuated ischemic inflammatory injury by suppressing endoplasmic reticulum stress-related signaling. Chen [111] further revealed that ICA at 1, 5, 10, 20, 40, or 80 μM protected N2a neuronal cells during OGD/reperfusion (with 10 μM showing the greatest improvement in cell viability) and inhibited neuronal apoptosis by downregulating PKM2 and reducing pro-apoptotic signaling. Additionally, pretreatment with ICA at 10 or 30 mg/kg twice daily for 3 consecutive days before 2 h of MCAO and 24 h of reperfusion was shown to inhibit IκB-α degradation and NF-κB activation, increase PPARα and PPARγ expression, reduce infarct volume and inflammatory signaling, and exert neuroprotective effects against cerebral ischemia–reperfusion injury [112]. It also reduced IL-1β, IL-6, and TNF-α expressions by inhibiting the IRE1/XBP1s signaling pathway, thereby mitigating I/R-injury-induced endoplasmic reticulum (ER)-stress-mediated inflammation [113]. Another study demonstrated that therapeutic hypothermia (TH) combined with ICA enhanced the modulation of apoptotic/inflammatory factor expression and activation of PPARs/Nrf2/NF-κB and JAK2/STAT3 pathways using mild hypothermia [114], improving its ameliorative effects on infarct volume, neurological deficits, and cell death, and thereby providing superior neuroprotection against IS.

In summary, ICA, a traditional Chinese medicine, exhibits unique advantages in IS treatment and can compensate for the limitations of existing drugs.

**Table 2:** The anti-inflammatory mechanisms and restorative effects of ICA.

Disease	Key Mechanisms of ICA	Therapeutic Effects	References
Hippocampal neuroinflammation	↓ HMGB1-RAGE, TLR4/NF-κB, and p38 MAPK; ↑ PI3K/AKT; microglia M2 polarization; ↓ NLRP3	Improves cognition, depression, and epilepsy outcomes	[75,76,77,78,79,80,81,82,83]
SCI	↓ IL-1β/TNF-α/iNOS and YAP signaling; ↑ Nrf2/HO-1	Motor recovery, reduced scar formation, and axon regeneration	[89,90,91,92]
MS/EAE	↓ NF-κB, iNOS, AKT, ERK, p38, and NLRP3; ↑ ERβ and GR	Myelin protection and immune balance	[96,97,98]
Neuropathic pain	↑ SIRT1 and Bcl-2; ↓ TNF-α, IL-6, Bax, and TRPV1/NR2B	Analgesic and neuroprotective	[102,103]
TBI	↓ NF-κB/MAPK, COX-2, and IL-1β; ↑ histone acetylation	Cognitive and neurological recovery	[106,107]
IS	↑ PPARα/γ and Nrf2; ↓ NF-κB, ER stress, and IL-1β	Neuroprotection and infarct reduction	[111,112,113,114,115]

**↓** indicates downregulation or inhibition; **↑** indicates upregulation or activation. Abbreviations: SCI, spinal cord injury; MS, multiple sclerosis; EAE, experimental autoimmune encephalomyelitis; TBI, traumatic brain injury; IS, ischemic stroke.

### 2.4. The Role of ICA in the Digestive System

Inflammatory bowel disease (IBD) faces treatment challenges due to drug limitations, while ICA targets intestinal inflammation for relief; similarly, non-alcoholic fatty liver disease (NAFLD) lacks effective drugs, but ICA shows promise by combating liver inflammation and injury, offering new therapeutic hope.

#### 2.4.1. Inflammatory Bowel Disease

IBD is a global health concern with increasing incidence, including Crohn’s disease (CD) and ulcerative colitis (UC), both of which are chronic relapsing intestinal inflammatory diseases. CD causes transmural inflammation that affects any gastrointestinal tract part, while UC is limited to colonic mucosal inflammation [115]. Various drugs, including corticosteroids, aminosalicylates, antibiotics, adjuvant therapies, and immunosuppressants, are used for IBD treatment, but all have adverse effects, such as high toxicity and drug resistance. Conventional courses temporarily relieve symptoms but fail to achieve long-term remission [116].

**Figure 4:**
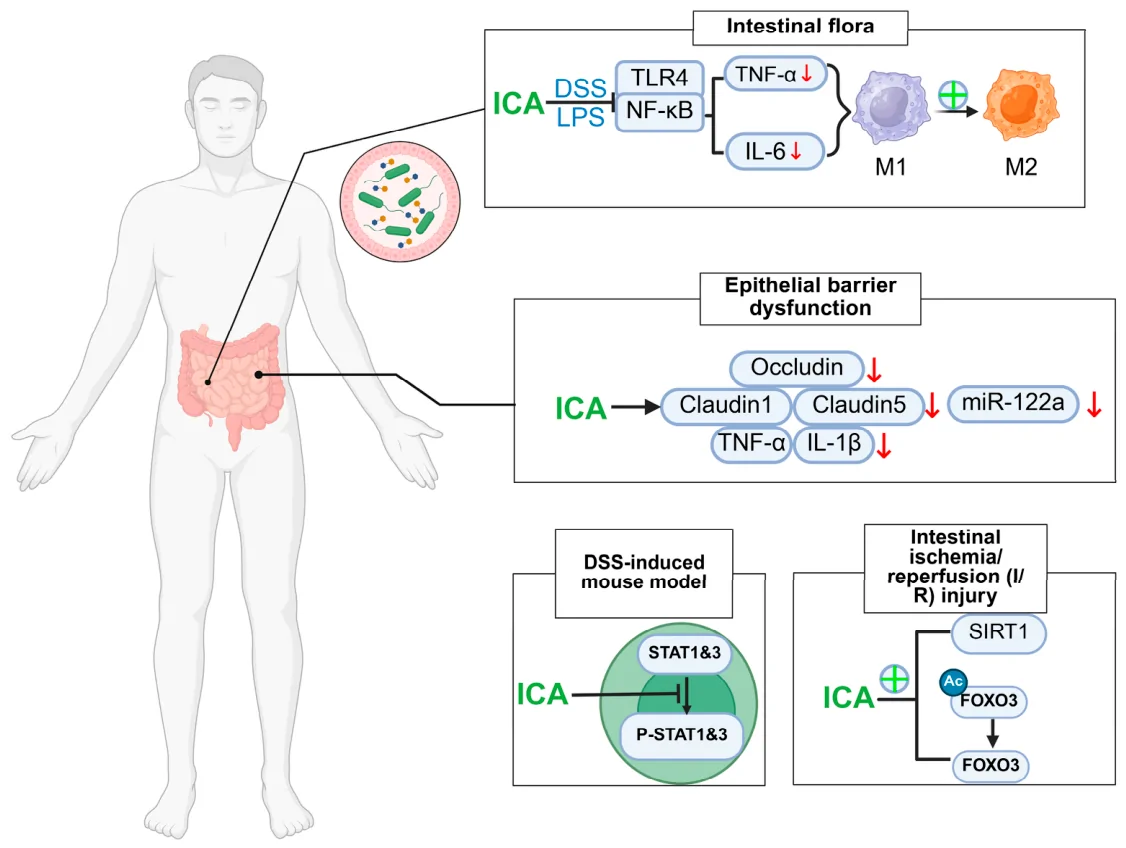
The mechanism of action of ICA on inflammatory bowel disease. This diagram integrates the multi-targeted mechanisms of ICA in alleviating IBD into a convergent regulatory framework centered on intestinal immune–microbial homeostasis. ICA simultaneously operates at three interconnected levels: (1) microbial regulation—restoring intestinal flora composition and abundance, which secondarily dampens TLR4/NF-κB-driven inflammatory cascades; (2) immune reprogramming—promoting anti-inflammatory M2 macrophage polarization while suppressing STAT1/STAT3-mediated Th1/Th17 responses, thereby shifting the mucosal immune milieu from a pro-inflammatory state toward a tolerogenic state; and (3) barrier restoration—reinforcing tight junction protein expression and SIRT1/FOXO3-dependent cytoprotection against epithelial injury. The functional interdependence of these layers—whereby microbial normalization reduces the luminal TLR4 ligand burden, immune polarization stabilizes epithelial integrity, and barrier repair prevents translocation-driven inflammation—explains the sustained efficacy of ICA in chronic colitis models. Red downward arrows (↓) indicate downregulation or inhibition; T-shaped bars (⊥) indicate inhibition or blockade; plus symbols (⊕) indicate promotion or positive regulation. Created in BioRender. Weifeng, L. (2026) https://BioRender.com/j6qhvux.

IBD pathogenesis is closely linked to intestinal flora imbalance, with changes in flora structure activating inflammatory signaling pathways and releasing inflammatory factors [117]. Comprehensive alterations in bacterial species, numbers, and distribution are closely associated with IBD intestinal inflammation development. ICA exerts anti-inflammatory effects by regulating intestinal flora abundance and composition, restoring histological structure and intestinal barrier function, inhibiting NF-κB signaling-pathway-mediated TNF-α and IL-6 expressions, suppressing TLR4 and NF-κB inflammatory signaling pathway activation by dextran sulfate sodium (DSS) or lipopolysaccharide (LPS), and switching the macrophage phenotype from M1 to M2, significantly reducing colonic inflammation and ensuring intestinal barrier function to prevent enteritis [118,119]. A study [120] demonstrated that ICA reduces the expression levels of the tight junction proteins Occludin, Claudin1, and Claudin5; attenuates epithelial barrier function impairment in the colon by inhibiting inflammatory factors TNF-α and IL-1β; decreases miR-122a expression; and alleviates colonic oxidative stress. In a DSS-induced mouse model, oral ICA at 3 or 10 mg/kg/day inhibited STAT1 and STAT3 phosphorylation in CD4+ T cells and suppressed Th1/Th17 responses, thereby exerting a therapeutic effect on experimental colitis [121]. Additionally, ICA pretreatment at 30 or 60 mg/kg in rats before intestinal ischemia/reperfusion and at 25, 50, or 100 μg/mL in Caco-2 cells before hypoxia/reoxygenation activated silent information regulator 1 (SIRT1) and promoted forkhead box protein O3 (FOXO3) deacetylation, thereby protecting intestinal epithelial cells from I/R-associated injury in vivo and in vitro [122]. Given enteritis’s multifactorial nature, ICA utilizes its multi-junction and multi-pathway anti-inflammatory effects to exert therapeutic effects on enteritis, overcoming the side effect limitations of current drugs (Figure 4).

#### 2.4.2. Liver Diseases

With improved living standards and unhealthy dietary habits, NAFLD has emerged as the most prevalent chronic liver disease globally, encompassing non-alcoholic fatty liver (NAFL), non-alcoholic steatohepatitis (NASH), cirrhosis, and hepatocellular carcinoma (HCC). Patients with NAFLD are more likely to die from extrahepatic complications, primarily cardiovascular disease, than from primary liver lesions [123]. Currently, no pharmacological treatments are approved for NAFLD, although vitamin E and pioglitazone have demonstrated some efficacy. However, vitamin E increases prostate cancer risk, while pioglitazone causes fluid retention and bone mineral density loss. Lifestyle interventions focusing on dietary calorie restriction and exercise remain the primary therapeutic approach [124]. The development of NAFLD is closely associated with lipid accumulation, oxidative stress, endoplasmic reticulum stress, and lipotoxicity.

In NASH pathogenesis, hepatic ferroptosis precedes other cell death types and triggers an inflammatory response [125]. Therefore, inhibiting ferroptosis may be crucial for preventing NASH progression and related liver injury. Choi [126] demonstrated that ICA at 50 or 100 mg/kg inhibited ferroptosis via the Nrf2-xCT/GPX4 pathway in a methionine choline deficiency (MCD)-diet-induced NASH mouse model, thereby attenuating NASH. Additionally, Zhao [127] found that ICA played a key role in preventing NASH progression and related liver injury by upregulating miR-206, which regulated the NF-κB and MAPK pathways, exerting a protective effect against NAFLD.

Hepatic ischemia–reperfusion (IR) injury is a major complication of liver transplantation that severely affects patient prognosis. Icaritin (ICT), a major plasma metabolite of ICA, has been shown to attenuate hepatic IR injury in mice through anti-inflammatory, anti-oxidative stress, and anti-autophagy effects. ICT significantly increased the expression ratios of p-PI3K/PI3K, p-AKT/AKT, and p-mTOR/mTOR proteins in hepatocytes in an animal model of hepatic IR injury and an oxygen-glucose deprivation/reoxygenation (OGD/R) cell model, reducing necrosis/apoptosis levels and inflammation-related cytokine content [128]. Shen [129] found that oral ICA at 25 or 50 mg/kg for 7 consecutive days before APAP administration attenuated acetaminophen (APAP)-induced acute liver injury by reducing inflammatory responses, oxidative stress, and hepatocyte apoptosis through the Nrf2, NF-κB, and p53 pathways; importantly, surface plasmon resonance analysis identified S100A9 as a direct ICA-binding target with an equilibrium dissociation constant of 1.14 μM. In summary, ICA exhibits hepatoprotective effects, offering an alternative approach to compensate for the lack of effective therapeutic drugs for NAFLD.

### 2.5. The Role of ICA Against Urinary Inflammation

Renal diseases and nonbacterial prostatitis face treatment limitations due to side effects or unclear etiology, whereas ICA shows multi-targeted efficacy by modulating Nrf2/NF-κB pathways; reducing fibrosis, oxidative stress, and apoptosis; and inhibiting PI3K–AKT signaling, offering a promising alternative for these conditions.

#### 2.5.1. Nephritis

Renal disease represents a prevalent non-infectious condition. Chronic kidney disease (CKD), for instance, is characterized by progressive deterioration leading to renal failure, requiring dialysis or kidney transplantation. However, neither treatment cures the disease, resulting in high mortality and disease burden associated with kidney failure [130]. Current medications tailored to specific disease conditions include antihypertensive drugs (e.g., ACEI/ARB), immunosuppressants (e.g., glucocorticosteroids), and diuretics (e.g., furosemide), yet their side effects, such as hyperkalemia, acute kidney injury, and cough induced by RAS inhibitors (ACEI/ARB), are pronounced.

For various nephritis types and kidney injuries, Ding [131] and Wang [132] observed that in unilateral ureteral obstruction (UUO) mice, ICA significantly improved renal function, reduced tubular injury, and decreased fibrotic lesions by alleviating Nrf2-mediated mitochondrial dysfunction, blocking pro-inflammatory cytokine IL-1β release, and exerting anti-renal mesangial inflammation and anti-tubulointerstitial fibrosis effects. In immunoglobulin A nephropathy (IgAN) rats, ICA administered via gavage at 10 mg/kg/day for 42 days markedly ameliorated glomerular fibrosis and renal injury by inhibiting NF-κB nuclear translocation and NLRP3 inflammasome activation [133]. Su [134] also reported that ICA at 10 mg/kg/day for 8 weeks effectively alleviated lupus nephritis in MRL/lpr mice by reducing anti-dsDNA antibody levels and renal immune-complex deposition and by suppressing NF-κB activation, macrophage infiltration, NLRP3 inflammasome activation, and downstream IL-1β production.

Xie [135] demonstrated that ICA at 30 or 60 mg/kg for 3 consecutive days before cecal ligation and perforation (CLP) reduced sepsis-associated mortality and acute kidney injury by suppressing renal oxidative damage, inflammatory responses, apoptosis, and vascular permeability, with the 60 mg/kg dose generally producing a greater renoprotective effect. Zhao [136] further revealed that ICA at 100 or 200 mg/kg/day markedly improved renal function in adenine-induced CKD rats in a dose-dependent manner by activating AMPK and regulating the AMPK/SIRT1/NF-κB and AMPK/ACC signaling pathways, thereby attenuating inflammatory responses, oxidative stress, and renal fibrosis. In a chronic tubulointerstitial nephritis (CTIN) model, Hou [137] found that ICA at 20 mg/kg/day, alone or in combination with selenomethionine (SeMet) at 0.4 mg/kg/day, attenuated renal dysfunction, pathological injury, apoptosis, and tubulointerstitial fibrosis by blocking TLR4/NF-κB signaling; in vitro, 2 μg/mL ICA combined with 2 μM selenomethionine produced the strongest protective effect against cyclosporine A- and ochratoxin A-induced injuries in renal tubular epithelial cells.

ICA also demonstrates significant efficacy in treating drug-induced nephropathy. During cisplatin chemotherapy, which often causes acute kidney injury in cancer patients, ICA reduced cisplatin-mediated nephrotoxicity by improving renal oxidative status; inhibiting subsequent NF-κB activation, inflammatory cascades, and apoptosis; and correcting abnormal apoptosis-related protein expression [138,139]. Qi [140] reported that ICA administered intragastrically at 150 mg/kg/day for 6 weeks reduced renal inflammation and functional injury in streptozotocin (STZ)-induced diabetic nephropathy (DN) mice by suppressing TLR4/NF-κB signaling and improving renal antioxidant defenses. Mishra [141] showed that oral ICA at 100 mg/kg/day for 2 consecutive weeks attenuated methotrexate (MTX)-induced renal injury in rats by restoring antioxidant enzyme activity, reducing lipid peroxidation, suppressing inflammatory responses and apoptosis, and activating the Nrf2/HO-1 pathway. Additionally, ICA protected against cyclophosphamide (CTX)-induced mouse kidney injury via the Keap1–Nrf2 pathway [142]. Zheng [143] also demonstrated that ICA attenuated cadmium (Cd)-induced renal injury in rats through antioxidant, anti-apoptotic, and anti-inflammatory mechanisms.

**Figure 5:**
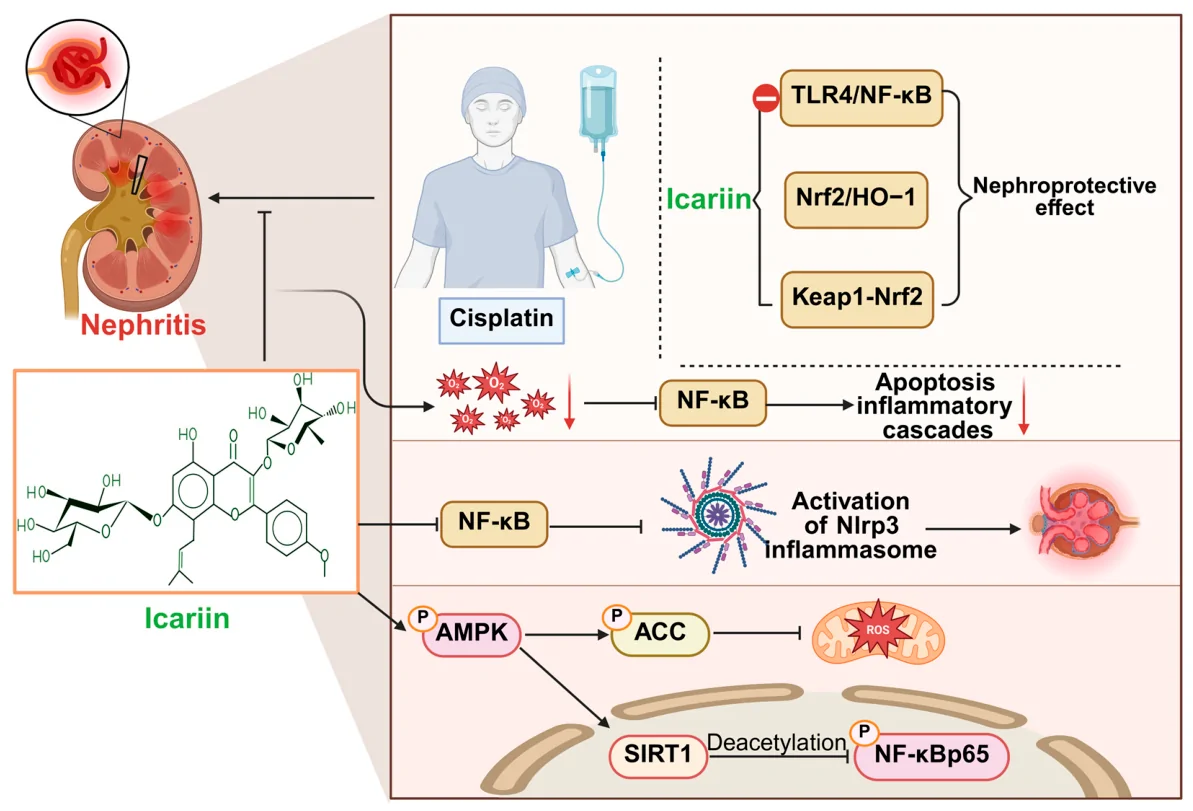
The therapeutic effects and mechanisms of ICA on kidney injuries. This figure delineates the nephroprotective and therapeutic effects of ICA across diverse renal diseases, including nephritis and drug-induced nephropathy. ICA enhances renal function and mitigates injuries by modulating key molecular pathways: it inhibits the TLR4/NF-κB signaling to reduce inflammation, activates Nrf2/HO-1 and Keap1-Nrf2 pathways for antioxidant and anti-inflammatory effects, and regulates AMPK/SIRT1/NF-κB and AMPK/ACC pathways to attenuate inflammatory responses and oxidative stress, thereby impeding renal fibrosis progression. These mechanisms collectively demonstrate ICA’s efficacy in alleviating kidney damage across various experimental models, affirming its potential as a therapeutic agent for renal protection. Red downward arrows (↓) indicate downregulation or reduction; prohibition symbols (Ө) and T-shaped bars (⊥) indicate inhibition or blockade. Created in BioRender. Weifeng, L. (2026) https://BioRender.com/0qvp0f6.

In summary, ICA has demonstrated consistent nephroprotective efficacy across diverse renal injury models (Figure 5). A recurring mechanistic theme emerges: ICA protects renal tissue by simultaneously suppressing NF-κB-mediated inflammatory cascades and activating Nrf2/HO-1/AMPK-dependent antioxidant and metabolic repair pathways. This dual-pronged defense—inflammation suppression coupled with endogenous cytoprotective pathway activation—operates across varied etiologies, including obstructive nephropathy (UUO), drug-induced injury (cisplatin, methotrexate), diabetic nephropathy, IgA nephropathy, and septic acute kidney injury. However, whether this convergent mechanism translates to consistent efficacy in human renal disease, where injury chronicity and comorbidity burden differ substantially from acute rodent models, remains to be established.

#### 2.5.2. Prostatitis

Prostatitis has an unclear etiology attributed to multiple factors, and its deep anatomical location makes clinical examination difficult, with infection as a common cause [144]. The main clinical presentations include acute and chronic bacterial prostatitis, nonbacterial prostatitis, and prostate pain. The pathogenesis of nonbacterial prostatitis, the most prevalent clinical syndrome, remains elusive, and due to the absence of a clear infectious etiology, antimicrobial treatment is ineffective [145]. For bacterial acute and chronic prostatitis, quinolones are the preferred agents, whereas for nonbacterial prostatitis, given the lack of a clear cause, a combination of antibiotics, antidepressants, α-blockers, counseling, non-steroidal anti-inflammatory drugs, sitz baths, and prostate massages is employed [146]. Moreover, the prostate’s special location complicates drug delivery, prompting continuous searches for novel and effective treatments.

Xu [147,148,149] found that the combination of ICA and curcumol inhibits prostate cancer progression by modulating the immune response and metabolism and mediating the interaction of intestinal flora with the DNMT1/IGFBP2 axis; affects lipid metabolism in prostate cancer cells by regulating the miR-7/mTOR/SREBP1 signaling pathway; and reverses docetaxel (DTX) resistance in prostate cancer (PCa) treatment by inhibiting the PI3K–AKT signaling pathway and the Warburg effect, thereby overcoming DTX-induced resistance in PCa. Sheng [150] also suggested that the drug combination induces iron death in prostate cancer cells by inhibiting the Nrf2/HO-1 signaling pathway, offering a potential novel iron death inducer for prostate cancer therapy. Thus, ICA–CUR acts on prostate cancer through multiple pathways.

Additionally, ICA effectively prevents benign prostatic hyperplasia (BPH) induced by metabolic syndrome (MetS) through antiproliferative, pro-apoptotic, antioxidant, and anti-inflammatory effects, as well as activation of the AMPK signaling pathway [151]. Another study demonstrated that co-loading ICA onto a carrier of a neutral homogeneous polysaccharide (EPSN-1) isolated from Epimedium shorticornum improved its biopharmacological properties, such as solubility and stability, thereby enhancing intestinal absorption. EPSN-1 also maintained intestinal microenvironment balance, enhancing the anti-BPH therapeutic effect [152].

Overall, ICA demonstrates convergent anti-inflammatory and anti-proliferative efficacy in non-bacterial prostatitis models and prostate cancer contexts, acting through NF-κB suppression, PI3K–AKT inhibition, and AMPK pathway activation. The consistency of these mechanistic targets across benign inflammatory and malignant conditions suggests a shared pathological signaling framework that ICA is uniquely positioned to address. Nevertheless, quantitative pharmacokinetic data supporting adequate ICA distribution to the prostatic compartment are currently lacking, and the predictive value of available preclinical models for the clinically heterogeneous and often non-infectious prostatitis syndromes remains uncertain.

### 2.6. The Role of ICA in the Motor System

Common musculoskeletal diseases such as osteoarthritis, rheumatoid arthritis, and osteoporosis arise from complex mechanisms with limited treatment options. ICA emerges as a promising agent by modulating inflammation via multiple targets, enhancing cartilage/bone repair, and inhibiting osteoclast activity.

#### 2.6.1. Osteoarthritis

Osteoarthritis (OA) is a chronic progressive disease characterized by degenerative cartilage loss, with a high prevalence and being a leading cause of disability. OA presents as a combination of factors, primarily joint pain, stiffness, and functional limitation, with knee lesions being the most common clinical manifestation globally [153]. Treatment typically combines non-pharmacological therapies, pharmacological interventions, and surgical procedures. Commonly used drugs, such as nonsteroidal anti-inflammatory drugs (NSAIDs) and cyclooxygenase-2 inhibitors (COX-2 inhibitors or coxibs), are associated with side effects, including gastrointestinal bleeding risk and cardiovascular safety concerns. No drugs can alter OA’s pathological progression; current treatments focus on symptom relief and recovery promotion [154]. OA and rheumatoid arthritis (RA) have different pathogeneses but similar inflammatory joint features.

Through network pharmacology and molecular docking technology, Zhang [155] found that ICA can inhibit the inflammatory response, chondrocyte apoptosis, and extracellular matrix degradation in knee osteoarthritis (KOA), promoting cartilage repair via multiple targets and pathways. Li [156] demonstrated that ICA treats KOA by regulating pain-related nerves and relieving pain. Studies have shown that ICA acts through multiple signaling pathways. Zhai’s study [157] revealed that ICA promotes bone formation by regulating the PI3K–AKT–eNOS–NO–cGMP–PKG signaling pathway, while Wu [158] and Wei [159] illustrated that ICA enhances osteogenic differentiation of rat bone marrow mesenchymal stem cells (BMSCs) through the ERK, p38, and JNK MAPK signaling pathways, as well as the ERα-Wnt/β-catenin signaling pathway. Liu [160] indicated that ICA may also play a protective role by inhibiting the synthesis of NO and MMP, reducing extracellular matrix destruction. Additionally, Zeng [161] showed that ICA at 5, 10, and 20 μM in IL-1β-stimulated human SW1353 chondrosarcoma cells, with 10 and 20 μM showing clear inhibition of MMP-13 and 20 μM producing no adverse effect on cell viability, reduces cartilage degradation by inhibiting MMP-13, phosphorylated p38, phosphorylated JNK, and β-catenin; intra-articular ICA at 20 μM was also used in a rat osteoarthritis model. Zu [162] implied that ICA exerts an anti-OA effect in in vitro and in vivo OA models by inhibiting cellular pyroptosis mediated through the NLRP3/caspase-1 signaling pathway; rat chondrocytes were exposed to 1, 2.5, 5, 10, 20, or 40 μM ICA, with the anti-pyroptotic effects showing concentration dependence, whereas Wistar rats received an intra-articular injection of 0.3 mL ICA at 20 μM 2 weeks after MIA administration. Wang [163] also demonstrated that ICA may attenuate inflammatory injury by inhibiting the NF-κB/HIF-2α signaling pathway, thereby enhancing chondrocyte viability. Mi [164] further found that ICA inhibits the NF-κB signaling pathway to activate autophagy, thereby inhibiting inflammatory factor release and regulating the balance between autophagy and apoptosis in chondrocytes; ICA was tested at 0, 3, 5, 7, 10, and 20 μM for 24–72 h, with 10 and 20 μM producing comparable beneficial effects and 10 μM being selected for subsequent mechanistic experiments. Various studies have demonstrated that ICA exerts therapeutic effects on OA by reducing cartilage lesions and damage, promoting cartilage repair and osteogenesis through multiple targets and pathways, and overcoming the side effects of Western anti-inflammatory drugs.

#### 2.6.2. Rheumatoid Arthritis

Rheumatoid arthritis (RA) is a systemic autoimmune disease characterized by a chronic inflammatory process that can damage joints and extra-articular organs, including the heart, kidneys, lungs, digestive system, eyes, skin, and nervous system [165]. Current therapies, which combine non-pharmacologic and pharmacologic approaches with disease-modifying anti-rheumatic drugs (DMARDs), achieve rapid onset of action. The main drugs include non-steroidal anti-inflammatory drugs (NSAIDs) and glucocorticosteroids [166]. NSAIDs act quickly but are associated with numerous side effects, including gastrointestinal, cardiovascular, hepatic, renal, cerebral, and pulmonary complications. Glucocorticoid-related side effects are more widespread, affecting the musculoskeletal, endocrine, digestive, neuropsychiatric, cardiovascular, skin, ocular, and immune systems [7]. Flavonoids, phenolic acids, alkaloids, and triterpenoids in traditional herbs have been identified as the primary components for improving RA.

ICA exerts an inhibitory effect on monosodium urate (MSU)-induced gouty arthritis (GA) in rats by modulating the NF-κB/NALP3 signaling pathway, with 20, 40, and 80 mg/kg used in vivo and 1, 5, and 10 μmol/L used in vitro [167]. Additionally, ICA at 10–80 μM reduces cell proliferation and inflammation in RA patient-derived fibroblast-like synoviocytes (FLSs) by regulating the GAREM1/MAPK signaling pathway, thereby alleviating RA progression [168]. Chi [169] also found that oral ICA at 25 mg/kg for 20 days decreases Th17 cells and IL-17 production by suppressing STAT3 activation, while 10 μM ICA was used in Th17-polarization experiments. Wu [170,171] demonstrated that ICA inhibits the TNF-α-induced inflammatory response and survival of RA-FLSs by regulating the TRIB1/TLR2/NF-κB signaling pathway; ICA also modulates the miR-223-3p/NLRP3 signaling pathway to inhibit RA-FLS proliferation and inflammatory response while promoting their apoptosis. As evidenced by these findings, ICA holds significant potential for RA treatment, overcoming the side effects of hormonal anti-inflammatory drugs.

#### 2.6.3. Osteoporosis

Osteoporosis is a systemic disease marked by reduced bone mass per unit volume and impaired bone strength, predisposing bones to fracture. It is an insidious, progressive condition with three main clinical manifestations: pain, fracture, and deformity. Its etiology is multifactorial, primarily involving peak bone mass acquisition around age 30 and subsequent bone loss post-menopause until old age [172]. Therapeutic agents include anti-bone resorption drugs (e.g., estrogens, selective estrogen receptor modulators, calcitonin, bisphosphonates, and denosumab) and pro-bone formation agents such as teriparatide (parathyroid hormone), which is the only approved bone-forming drug in the U.S. that stimulates new bone formation, repairs structural defects, and increases bone density. However, anti-bone resorption drugs have high administration requirements and adverse effects, such as gastrointestinal issues, osteonecrosis of the jaw, and atypical femur fractures, while teriparatide has a maximum lifespan of 2 years, limiting its long-term use [173].

ICA shares core genes and KEGG signaling pathways with osteoporosis-related genes, participating in the regulation of inflammation, insulin resistance, apoptosis, and immune response. It can modulate signaling pathways such as PI3K–AKT, NF-κB, MAPK, and JNK. Some in vitro experimental data suggest that ICA may exert anti-osteoporosis effects by inhibiting the JNK/c-Jun signaling pathway [174]. Zhang [175] found that ICA may alleviate Glucocorticoid-Induced Osteoporosis (GIOP), with 5 μM used in vitro and 100 mg/kg used orally for 6–12 weeks in animal models. Zhai [176] implied that ICA at 0.01–20 μM could inhibit the nuclear translocation of the NF-κB p65 protein, promote the gene expression of type II collagen (Col-II) and aggregated proteoglycan (Aggrecan) in chondrocytes, reduce NF-κB expression, and significantly decrease IL-6 and TNF-α levels in an LPS-induced inflammation model, thereby enhancing chondrocyte proliferation and promoting phenotypic gene expression while inhibiting chondrocyte degeneration. Sun [177] demonstrated that ICA promotes osteoblast proliferation and differentiation through estrogen-receptor-mediated regulation of the OPG/RANKL ratio, with 50 ng/mL identified as the optimal concentration. Liu [178] and Hsieh [179] confirmed that ICA inhibits osteoblast and osteoclast differentiation in vitro; reverses LPS-induced bone loss by downregulating miR-34c and inhibiting JNK, p38, and NF-κB signaling pathways; and blocks LPS-induced osteoclastogenesis by inhibiting p38 and JNK signaling pathway activation. Furthermore, Liu [180] showed that ICA may promote osteoblast proliferation and differentiation under overload by activating the RUNX2 promoter and Wnt/β-catenin signaling pathway. Despite its potential, ICA’s bioavailability limits its efficacy. Mohammadzadeh’s study [181] indicated that nanoplatforms can significantly enhance bone regeneration through multidimensional modulation, including targeted cellular differentiation guidance, inflammatory response modulation, and optimized drug release properties. These nanocarriers promote optimal osteogenic effects by enhancing alkaline phosphatase activity, upregulating RANKL expression, and promoting calcified nodule formation in bone tissue via the BMP-2/Smad4 signaling pathway. Collectively, these findings reveal a functionally convergent mechanism in which ICA promotes bone homeostasis through the co-regulation of two opposing processes: osteoblast differentiation and survival (via PI3K-AKT, Wnt/β-catenin, and RUNX2) and osteoclast suppression (via OPG/RANKL, NF-κB, and MAPK/JNK inhibition). This coordinated dual action—simultaneously stimulating bone formation and restraining bone resorption—distinguishes ICA from anti-resorptive monotherapies and positions it as a candidate for multi-faceted osteoporosis intervention, subject to resolution of its bioavailability limitations.

## 3. Bridging Preclinical Evidence to Clinical Translation

### 3.1. Translational Bottlenecks: Why ICA Has Not Reached the Clinic

Despite accumulating preclinical evidence supporting the anti-inflammatory and multi-target pharmacological activities of ICA, its clinical translation remains constrained by several interconnected barriers. Natural product-based drug discovery has regained considerable attention in recent years; however, the transition from an experimental bioactive compound to a clinically usable therapeutic agent requires rigorous evidence concerning pharmacokinetics, safety, pharmaceutical quality, and reproducible supply [182].

**Figure 6:**
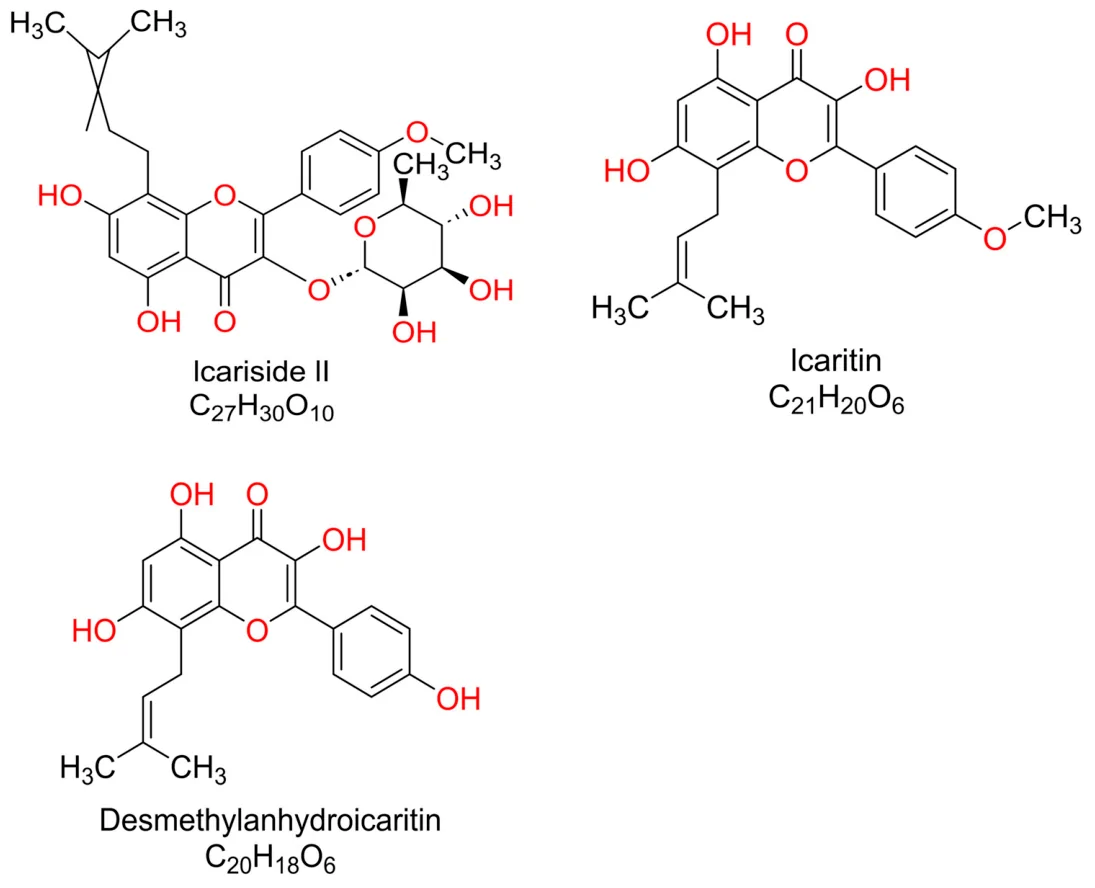
Structural features of icariside II, icaritin and desmethylicaritin, the primary intestinal metabolites of ICA, are derived via sequential deglycosylation and demethylation of parent ICA. These metabolites retain the flavonoid backbone and prenyl side chain of ICA, while modifications to sugar moiety and aromatic substituents alter polarity, membrane permeability and biological activity. In the structures, oxygen atoms (hydroxyl, carbonyl, and methoxy groups) are highlighted in red to facilitate comparison of the structural differences among the three metabolites.

First, ICA exhibits poor aqueous solubility and limited membrane permeability, which contribute substantially to its low oral bioavailability. Its pharmacokinetic behavior is further complicated by extensive intestinal and presystemic metabolism. Human intestinal microflora can rapidly transform ICA into several structurally related metabolites, including icariside II, icaritin, and desmethylicaritin. In this review, the term “major metabolites” refers to repeatedly identified and pharmacologically relevant biotransformation products of ICA rather than necessarily the most abundant circulating metabolites under every dosing condition. The chemical structures of icariside II, icaritin, and desmethylicaritin are displayed in the following figure (Figure 6). Structurally, icariside II is formed mainly by removal of the glucose residue from ICA; further deglycosylation yields the aglycone icaritin, and subsequent O-demethylation can generate desmethylicaritin. These sequential structural changes markedly alter polarity, membrane permeability, and systemic exposure and may contribute to the pharmacological activity observed after oral ICA administration. In rats, approximately 91.2% of orally administered ICA was converted to icariside II, and the Cmax and AUC0–t of icariside II were approximately 3.8- and 13.0-fold higher, respectively, than those of the parent ICA, whereas only 0.4% of ICA was converted to icariside II after intravenous administration. These findings indicate that systemic pharmacological activity after oral ICA administration may depend substantially on metabolite exposure rather than on the parent compound alone [183,184].

Second, intestinal metabolism constitutes a key determinant of ICA exposure and is a major cause of the low systemic level of parent ICA. Experimental studies indicate that ICA is extensively converted before or during intestinal absorption, and icariside II frequently represents an important intermediate in this metabolic sequence. Most ICA is converted to icariside II before intestinal absorption, and the subsequent formation of icaritin and desmethylicaritin further accounts for the discrepancy between the low systemic exposure of native ICA and its extensive in vivo pharmacological effects [185]. In addition, intestinal and hepatic phase II metabolism, particularly glucuronidation, may further contribute to presystemic clearance through UDP–glucuronosyltransferase (UGT)-mediated conjugation [186]. The magnitude of the formulation effect further illustrates the extent to which poor absorption limits native ICA. In rats that received total flavonoids of *E. brevicornu* Maxim., nanosuspension and cyclodextrin formulations increased the relative bioavailability of ICA and icariside II to approximately 228–295% of that obtained with the crude preparation, whereas a solid-dispersion formulation increased the relative bioavailability of ICA to approximately 416% [184]. These data support pharmaceutical formulation as an important determinant of systemic ICA exposure and further confirm that ICA pharmacology should be interpreted based on its metabolites rather than the parent compound alone.

Third, the absence of high-quality clinical evidence remains the most significant barrier preventing ICA from entering routine clinical practice. Despite extensive cellular and animal studies, human pharmacokinetic parameters, dose–exposure relationships, therapeutic concentration ranges, long-term safety profiles, and randomized clinical efficacy data remain largely unavailable. Recent reviews have therefore emphasized that future ICA development should shift from predominantly mechanistic pharmacology toward standardized pharmaceutical development, systematic pharmacokinetic evaluation, and rigorous clinical investigation [187,188].

Collectively, poor aqueous solubility and membrane permeability, extensive intestinal and presystemic metabolism, dependence on active metabolites, and the lack of human dose–exposure and efficacy data largely explain why ICA remains at the preclinical stage. Resolving these limitations will require integrated pharmacokinetic investigation, formulation optimization, toxicological assessment, standardized manufacturing, and well-designed early-phase clinical studies.

### 3.2. Strategies to Overcome Translational Barriers

To overcome these translational limitations, future ICA development should integrate formulation engineering, metabolite-guided pharmacokinetic research, structural optimization, and rigorous early-phase clinical evaluation. Among these approaches, improving oral bioavailability is an immediate priority because poor solubility and limited absorption directly restrict systemic exposure following oral administration [183].

Advanced drug-delivery systems have demonstrated substantial potential to improve ICA delivery. A colon-targeted alginate–chitosan microsphere system released only approximately 10% of loaded ICA in simulated gastric fluid but approximately 65.6% in simulated colonic fluid, while maintaining colon-associated microsphere retention for more than 12 h and reducing inflammatory injury in a rat model of colitis [189]. Cyclodextrin-based complexation produced an even greater pharmacokinetic improvement: complexation of ICA with hydroxypropyl-γ-cyclodextrin increased its aqueous solubility approximately 654-fold and its dissolution rate approximately 80-fold, while achieving nearly complete release within the first few minutes. In dogs, the complex increased ICA’s Cmax by approximately 5-fold and AUC0–120 by approximately 20-fold, prolonged the half-life from 0.68 to 6.38 h, reduced clearance from 11.17 to 0.65 L/h/kg, and increased the relative bioavailability by nearly 20-fold [190]. These quantitative findings demonstrate that formulation engineering can substantially modify ICA systemic exposure.

Disease-oriented delivery platforms may further expand the therapeutic potential of ICA. Encapsulation of ICA in β-cyclodextrin/amidated sodium alginate (β-CD-ALG) composites produced sustained release for up to 7 days; formulations containing 1, 5, and 10 μM ICA released approximately 71.5%, 80.6%, and 80.6% of their ICA contents, respectively, over this period and enhanced the osteogenic activity in MC3T3-E1 cells [191]. Similarly, icariin-functionalized nanodiamonds (ICA-NDs) prolonged ICA release for up to 4 weeks, and ICA (50 μg)-NDs produced greater increases in alkaline phosphatase activity, calcium deposition, and osteogenic gene expression than ICA (10 μg)-NDs [192].

Alternative administration routes may also improve tissue-specific delivery. In a traumatic brain injury (TBI) model, intradermal administration of an icariin/HP-β-cyclodextrin (HP-β-CD) inclusion complex at 0.4 mg/kg through the mystacial pad produced greater neurovascular protection than intranasal or intravenous administration, supporting the potential of the trigeminal epineurium–dura mater–brain pathway for brain-targeted ICA delivery [193].

Targeted and stimulus-responsive nanocarriers may provide additional advantages, although the chemical identity of the active compound must be clearly distinguished. A study showed that a multifunctional SiO_2_/icaritin–PDA–FA nanocomposite exploited folic acid-mediated tumor targeting and acidic intracellular conditions to promote controlled icaritin release and enhance low-temperature photothermal therapy. Therefore, this formulation should be discussed as an icaritin-based delivery strategy rather than direct evidence for ICA pharmacokinetic improvement [194].

In addition to formulation optimization, a metabolite-centered development strategy should be adopted. Because approximately 91.2% of orally administered ICA can be converted to icariside II in rats, and human intestinal microorganisms rapidly generate icariside II, icaritin, and desmethylicaritin, future studies should quantify parent ICA and its principal active metabolites when evaluating systemic exposure [184,185]. Such an approach may help explain the discrepancy between the relatively low circulating concentration of parent ICA and the broad pharmacological effects observed in vivo.

Structural modification also represents an important complementary strategy. Pharmaceutical technologies, absorption-enhancing approaches, and chemical modification may improve the solubility, membrane permeability, metabolic stability, tissue targeting, and overall drug-like properties of ICA and its derivatives [183,187]. However, improved in vitro activity or formulation performance should not be considered sufficient evidence of translational superiority unless accompanied by quantitative pharmacokinetic and toxicological evaluation.

Finally, clinical translation requires a stepwise development framework. Future studies should establish standardized Good Manufacturing Practice (GMP)-compatible production and quality-control procedures; conduct Good Laboratory Practice (GLP)-compliant repeated-dose, reproductive, and endocrine toxicity studies; and systematically characterize human absorption, distribution, metabolism, and excretion (ADME). Early human pharmacokinetic studies should quantify ICA, together with icariside II, icaritin, and other relevant metabolites, and establish Cmax, AUC, half-life, clearance, bioavailability, and dose proportionality values. Physiologically based pharmacokinetic models may subsequently support dose selection and translational prediction when developed and validated according to regulatory standards [195]. Only after these prerequisites are fulfilled should ICA proceed to phase I safety and pharmacokinetic trials, followed by proof-of-concept studies in clearly defined inflammatory disease populations.

## 4. Conclusions and Future Directions

In this review, we summarized ICA’s anti-inflammatory effects, exerted through multiple mechanisms, including regulation of inflammatory signaling pathways such as inhibition of NF-κB, MAPK, and related cascades; modulation of immune cell homeostasis, including restoration of Th17/Treg balance; activation of antioxidant defenses via Nrf2/HO-1 and related pathways; inhibition of apoptosis and pyroptosis; and protection of tissue-specific functions such as endothelial barrier integrity and promotion of osteogenic differentiation.

Although considerable progress has been made in elucidating the molecular mechanisms underlying ICA actions, the current evidence remains predominantly derived from cell-based assays and small-animal models. This highlights the pressing need for a more systematic translational research framework before ICA can be advanced to clinical application [196]. Future studies should first establish comprehensive human pharmacokinetic and ADME profiles, because ICA’s low oral bioavailability and the extensive biotransformation of its active metabolites remain major factors limiting its therapeutic translation. In parallel, rigorous GLP-compliant long-term safety studies, including chronic toxicity, reproductive toxicity, and endocrine safety evaluations, should be completed, together with standardized GMP-compatible manufacturing procedures and quality-control systems to ensure batch-to-batch consistency of pharmaceutical-grade ICA preparations. Furthermore, advanced pharmaceutical strategies, including nanocarrier-based delivery systems, cyclodextrin complexation, and other bioavailability-enhancing formulations, are expected to improve systemic exposure and maximize the therapeutic efficacy of ICA, thereby facilitating its subsequent clinical development. Ultimately, well-designed first-in-human studies, followed by randomized controlled clinical trials in carefully selected inflammatory diseases, will be indispensable to determine the efficacy, safety, and translational value of ICA. Successful integration of mechanistic pharmacology, pharmaceutical engineering, and evidence-based clinical investigation may enable ICA to evolve from a promising natural compound into a clinically applicable multi-target anti-inflammatory drug.

## Data Availability

No new data were created or analyzed in this study. Data sharing is not applicable to this article.

## Abbreviations

The following abbreviations are used in this review:

ACEIs	Angiotensin-Converting Enzyme Inhibitors
ADME	absorption, distribution, metabolism and excretion
ALI	Acute Lung Injury
AMPK	AMP-Activated Protein Kinase
APAP	Acetaminophen
ARB	Angiotensin II Receptor Blockers
ARDS	Acute Respiratory Distress Syndrome
AS cell	Atherosclerosis Cell
ASMCs	Airway Smooth Muscle Cells
BALF	Bronchoalveolar Lavage Fluid
BMSCs	Bone Marrow Mesenchymal Stem Cells
BLM	Bleomycin
CB1	Cannabinoid Receptor 1
CB2	Cannabinoid Receptor 2
CAD	Coronary Atherosclerotic Heart Disease
CD	Crohn’s Disease
CKD	Chronic Kidney Disease
CLP	Cecum Ligation And Perforation
COPD	Chronic Obstructive Pulmonary Disease
CRH	Corticotropin-Releasing Hormone
CRTH2	PGD2 Receptor 2
CTIN	Chronic Tubulointerstitial Nephritis
CTX	Cyclophosphamide
DCM	Diabetic Cardiomyopathy
DMARDs	Disease-Modifying Anti-Rheumatic Drugs
DN	Diabetic Nephropathy
EAE	Experimental Autoimmune Encephalomyelitis
eNOS	Endothelial Nitric Oxide Synthase
EPSN-1	Neutral Homogeneous Polysaccharide Isolated From Epimedium Shorticornum
FLS	Fibroblast-Like Synoviocytes
GIOP	Glucocorticoid-Induced Osteoporosis
GLP	Good Laboratory Practice
GMP	Good Manufacturing Practice
GRα	Glucocorticoid Receptor α
HAVSMCs	Arterial Vascular Smooth Muscle Cells
HCD	High Cholesterol Diet
HDA	High-Density Lipoprotein Biomimetic Carrier
HMGB1	High Mobility Group Box 1
HPA	Hypothalamic–Pituitary–Adrenal
HO-1	Heme Oxygenase-1
HP-β-CD	Hydroxypropyl-β-Cyclodextrin
HUVECs	Human Umbilical Vein Endothelial Cells
ICA	Icariin
IgAN	Immunoglobulin A Nephropathy
IPC	Icariin-Phospholipid Complex
I/R	Ischemia/Reperfusion
LDE	Low-Density Lipoprotein Biomimetic Carrier
LPS	Lipopolysaccharide
MAPK	Mitogen-Activated Protein Kinase
MCD	Methionine Choline Deficiency
MI	Myocardial Infarction
MS	Multiple Sclerosis
MSU	Monosodium Urate
MTX	Methotrexate
NAFLD	Non-Alcoholic Fatty Liver Disease
NASH	Non-Alcoholic Steatohepatitis
NF-κB	Nuclear Factor-κB
Nrf2	Nuclear Factor Erythroid 2-Related Factor 2
NSAIDs	Non-Steroidal Anti-Inflammatory Drugs
NP	Neuropathic Pain
OA	Osteoarthritis
OGD/R	Oxygen-Glucose Deprivation/Reoxygenation
Ox-LDL	Oxidized Low-Density Lipoprotein
PD-1	Programmed Death Protein-1
PF	Pulmonary Fibrosis
PGD2	Prostaglandin D2
PPARγ	Peroxisome Proliferator-Activated Receptor γ
RA	Rheumatoid Arthritis
RSV	Respiratory Syncytial Virus
SCI	Spinal Cord Injury
SIRT1	Silent Information Regulator 1
SHRs	Spontaneously Hypertensive Rats
T2DM	Type 2 Diabetes Mellitus
TBI	Traumatic Brain Injury
TGF-β	Transforming Growth Factor-β
TLR4	Toll-Like Receptor 4
UC	Ulcerative Colitis
UGT	UDP-glucuronosyltransferase
UUO	Unilateral Ureteral Obstruction
YAP	Yes-Associated Protein
